# Phosphatidic acid-mediated binding and mammalian cell internalization of the *Vibrio cholerae* cytotoxin MakA

**DOI:** 10.1371/journal.ppat.1009414

**Published:** 2021-03-18

**Authors:** Aftab Nadeem, Athar Alam, Eric Toh, Si Lhyam Myint, Zia ur Rehman, Tao Liu, Marta Bally, Anna Arnqvist, Hui Wang, Jun Zhu, Karina Persson, Bernt Eric Uhlin, Sun Nyunt Wai

**Affiliations:** 1 Department of Molecular Biology, Umeå University, Umeå, Sweden; 2 Umeå Centre for Microbial Research (UCMR), Umeå University, Umeå, Sweden; 3 Department of Clinical Microbiology, Umeå University, Umeå, Sweden; 4 Department of Medical Biochemistry and Biophysics, Umeå University, Umeå, Sweden; 5 Department of Biotechnology and Genetic Engineering, Kohat University of Science and Technology, Khyber Pakhtunkhwa, Pakistan; 6 Department of Microbiology, College of Life Sciences, Nanjing Agricultural University, Nanjing, China; 7 Wallenberg Centre for Molecular Medicine, Umeå University, Umeå, Sweden; 8 Department of Microbiology, School of Medicine, University of Pennsylvania, Philadelphia, Pennsylvania, United States of America; 9 Department of Chemistry, Umeå University, Umeå, Sweden; 10 The Laboratory for Molecular Infection Medicine Sweden (MIMS), Umeå University, Umeå, Sweden; University of Illinois, UNITED STATES

## Abstract

*Vibrio cholerae* is a noninvasive intestinal pathogen extensively studied as the causative agent of the human disease cholera. Our recent work identified MakA as a potent virulence factor of *V*. *cholerae* in both *Caenorhabditis elegans* and zebrafish, prompting us to investigate the potential contribution of MakA to pathogenesis also in mammalian hosts. In this study, we demonstrate that the MakA protein could induce autophagy and cytotoxicity of target cells. In addition, we observed that phosphatidic acid (PA)-mediated MakA-binding to the host cell plasma membranes promoted macropinocytosis resulting in the formation of an endomembrane-rich aggregate and vacuolation in intoxicated cells that lead to induction of autophagy and dysfunction of intracellular organelles. Moreover, we functionally characterized the molecular basis of the MakA interaction with PA and identified that the N-terminal domain of MakA is required for its binding to PA and thereby for cell toxicity. Furthermore, we observed that the *ΔmakA* mutant outcompeted the wild-type *V*. *cholerae* strain A1552 in the adult mouse infection model. Based on the findings revealing mechanistic insights into the dynamic process of MakA-induced autophagy and cytotoxicity we discuss the potential role played by the MakA protein during late stages of cholera infection as an anti-colonization factor.

## Author summary

*Vibrio cholerae* is the cause of cholera, an infectious disease causing watery diarrhea that can lead to fatal dehydration. The bacteria can readily adapt to different environments, such as from its natural aquatic habitats to the human digestive system. Recently, we reported a novel *V*. *cholerae* cytotoxin, MakA that functions as a potent virulence factor in *C*. *elegans* and zebrafish. Here we identified phosphatidic acid as a lipid target for MakA interaction with mammalian cells. This interaction promoted macropinocytosis resulting in the formation of an endomembrane-rich aggregate in intoxicated cells that ultimately lead to activation of autophagy. Importantly, data from bacterial colonization in a mouse infection model suggested that MakA might act as an anti-colonization factor of *V*. *cholerae*, presumably expressed during later stage(s) of infection. MakA might be explored as a new target for diagnostics and therapeutic developments against *V*. *cholerae* infections. Our findings will contribute to further understanding of the virulence, colonization and post-infection spread of *V*. *cholera*e.

## Introduction

*Vibrio cholerae*, the causative agent of the disease cholera, is an extracellular facultative human pathogen, with aquatic and intestinal life cycles [1,2]. *V*. *cholerae* is classified into more than 200 serogroups based on the O-antigen structures and the subgroups O1 and O139 are known to cause cholera [3]. *V*. *cholerae* O1 and O139 serogroups express the cholera toxin (CT), a main virulence factor, and the toxin co-regulated pilus (TCP), which are responsible for diarrhea and intestinal colonization, respectively. In order to initiate the disease, *V*. *cholerae* evades the host intestinal innate immune system, penetrates the small intestine mucus layer, adheres to the surface of microvilli, and produces a broad range of toxin(s) through the action of virulence-associated genes [4]. Recently we reported a novel *V*. *cholerae* cytotoxin, MakA (motility associated killing factor A), that functions as a potent virulence factor in *C*. *elegans* and zebrafish [5].

Regulation of host signaling pathways by bacterial pathogens is critical for colonization and replication within, or in the close vicinity of, eukaryotic host cells. To achieve the best possible colonization condition, many bacterial species have evolved a variety of molecular mechanisms that include direct delivery of effector proteins to the host cell membrane [6]. Eukaryotic cell membrane trafficking pathways include a series of highly dynamic endocytic, autophagic and secretory pathways [7]. Most intracellular bacteria use a special mechanism to invade non-phagocytic cells, characterized by induction of macropinocytosis, an endocytic pathway that involves actin-mediated membrane ruffling and engulfment that ultimately leads to the formation of macropinosomes [8–10]. Macropinocytosis is initiated via actin polymerization, upon hyper-stimulation of growth factor receptors leading to activation of phosphoinositide 3-kinase (PI3K) and small GTPases [11,12].

Phosphatidic acid (PA) is an important precursor for the biogenesis of other phospholipids and it constitute on average about 1–4% of the total phospholipid content of eukaryotic cells [13–15]. Several PA binding proteins found in mammalian cells including guanosine triphosphatases (GTPases), kinases, and phospholipases are reported and there is increasing evidence that PA is a second messenger that contributes to a wide variety of cell-signaling pathways [16,17]. In bacteria, such functions have not been extensively studied and only few bacterial PA-binding proteins have been identified so far. The cytoplasmic protein PA3911 in *P*. *aeruginosa* was shown to be a PA-binding protein involved in lipid homeostasis in the bacterial cells [18]. The *Vibrio parahaemolyticus* multivalent adhesion molecules (MAM7) has the ability to bind host PA directly and, therefore, attach to a wide range of host cell types, including epithelial cells, fibroblasts and macrophages [19]. In the current study, we report that the recently discovered *V*. *cholerae* cytotoxin, MakA, binds to a host cell via mechanisms involving an interaction to PA. This binding leads to macropinocytosis of the toxin that ultimately causes disruption of the intracellular organelles. In addition, we demonstrate that the MakA protein could induce autophagy and cytotoxicity in the target mammalian cells in a PA-dependent manner. Finally, we characterized the molecular basis of MakA interaction with PA.

## Results

### Effect of secreted MakA protein from *V*. *cholerae* on the tumor cell line

**Fig 1:**
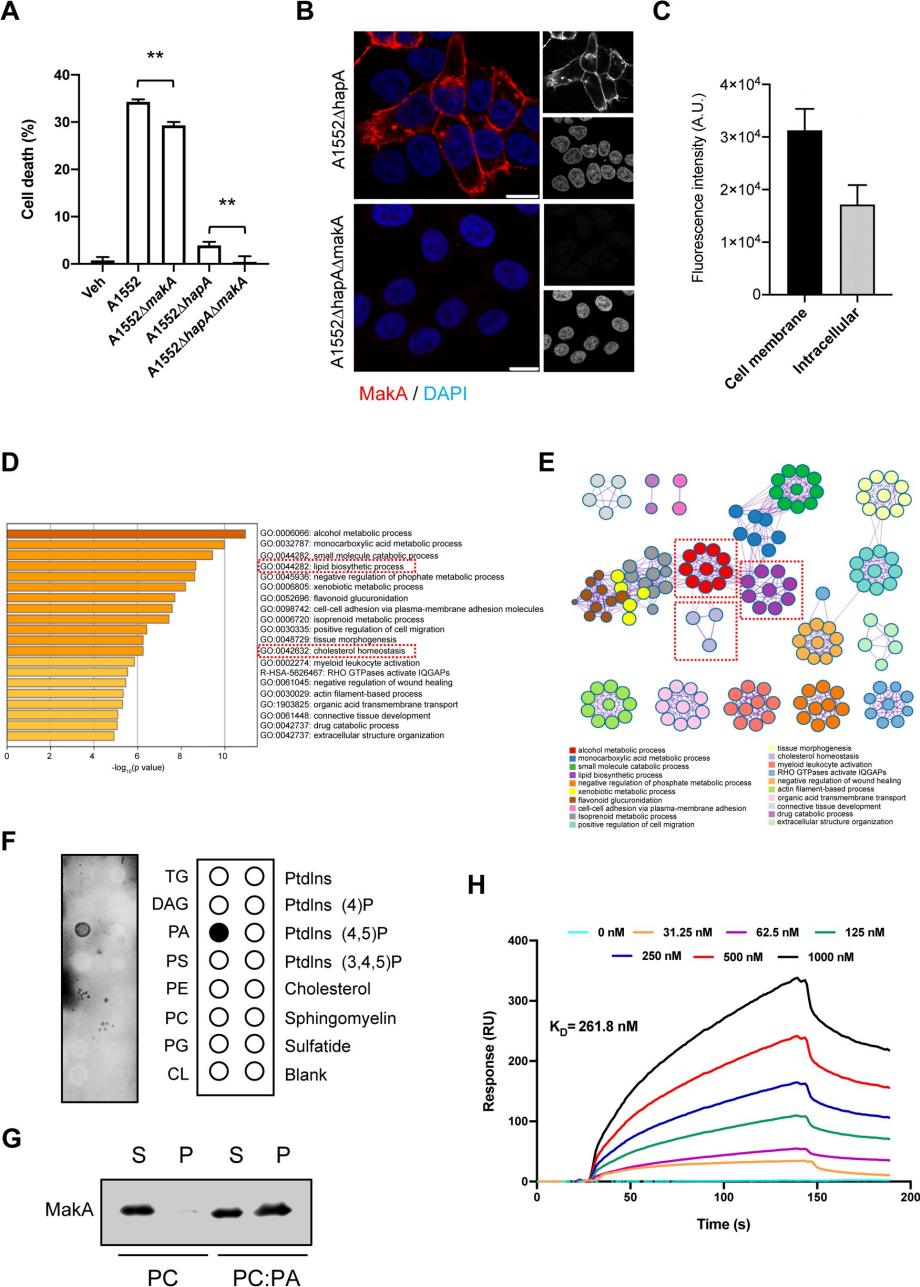
MakA contributes to host cell toxicity and it binds to PA. **(A)** HCT8 cell toxicity was detected by staining the cells with propidium iodide. Fluorescence values for propidium iodide was recorded on a plate reader. Data was normalized against cells treated with TritonX-100 (0.1%). Data are representative of three independent experiments; bar graphs show mean ± s.d. Significance was determined from replicates using a one-way analysis of variance (ANOVA) with Sidak’s multiple comparisons test. **p<0.01. **(B)** HCT8 cells treated with culture supernatants (10%) from A1552**Δ***hapA* or A1552**Δ***hapA*Δ*makA*. Uptake of MakA (red) was assessed by confocal microscopy using anti-MakA antiserum. Nuclei were counterstained with DAPI (blue). Scale bars, 10 μm **(C)** Histograms indicate cell membrane bound and intracellular uptake of MakA (n = 100 random cells). Data from two independent experiments is presented as mean ± s.e.m. **(D**) Top twenty gene ontology (GO) and reactome terms from Metascape express enrichment analysis using differentially expressed genes (log_2_FC≤1, p≥0.05) from HCT8 cells treated with MakA (500 nM, 48 h). The dotted rectangles (red) indicate gene sets involved in lipid signaling. **(E)** Network of enriched gene sets colored by ID. Threshold: 0.3 kappa score. Networks highlighted in dotted red boxes indicate gene networks involved in cellular metabolism or lipid signaling. **(F)** Membrane lipids strip precoated with indicated lipids were incubated with His-MakA (50 μg/mL) and probed with anti-His tag monoclonal antibodies. Lipid membrane to the right shows individual lipid species coated on the nitrocellulose membrane. The black dot indicates MakA bound lipid species. **(G)** Purified MakA (5 μg/mL) was incubated with PC or PC:PA (1:1) liposomes (200 μM) and then separated by centrifugation. Liposome-bound MakA was detected with anti-MakA antisera. Data are representative of two independent experiments. S = supernatant and P = pellet. **(H)** Surface plasmon resonance (SPR) assay showing direct binding of MakA (0 to 1000 nM, as obtained by serial two-fold dilutions) to PA:PC (1:1) liposomes immobilized on an SPR sensor chip L1 (turquoise line with 0 nM is buffer control). Control flow cell background was subtracted from the experimental cell before final data processing. The K_D_ values were calculated by using the BioLogic scrubber 2 software. All data underlying the findings reported are shown in **S1 Data**.

Recently we reported a novel flagella-mediated secreted *V*. *cholerae* cytotoxin, MakA that functions as a potent virulence factor in *C*. *elegans* and zebrafish [5]. Although the MakA protein caused lethal effect on *C*. *elegans* and zebrafish, it has remained unclear how the MakA protein may interact with mammalian host cells. To determine if the MakA protein in *V*. *cholerae* is potentially involved in modulating cellular process of host cells, we isolated culture supernatants from the wild type *V*. *cholerae* El Tor O1 strain A1552 and its isogenic Δ*makA* mutant and investigated possible effects on the human colon tumor cell line HCT8. Both supernatants caused dramatic changes in host cell morphology in comparison with vehicle-treated cells (**S1A Fig**). In previous studies, we demonstrated that the most abundant secreted protein from this *V*. *cholerae* strain is hemagglutinin protease A (HapA) [20]. In addition, earlier studies showed that HapA could perturb the paracellular barrier function in epithelial cells by degrading the tight junction protein, occludin [21]. The rounding and detachment of host cells treated with culture supernatant from the wild-type *V*. *cholerae* strain A1552 might be due to the dominant action of HapA protease activity. We therefore, constructed Δ*hapA* and Δ*hapA*Δ*makA* mutants and treated the HCT8 cells with supernatants isolated from these two mutant strains. In comparison with the cells treated with culture supernatant from the wild type strain A1552, the cells treated with the culture supernatant from the Δ*hapA* mutant clearly showed reduced cell rounding effect (**S1A Fig**). The cytotoxic effect was further reduced in the cells treated with supernatant from the Δ*hapA*Δ*makA* mutant in comparison with the cells treated with supernatant from the Δ*hapA* mutant strain (**Fig 1A**) suggesting that secreted MakA in the supernatant from *makA*^*+*^ bacteria also contributed to the cytotoxic phenotype of the target cells. Next, we tested if the secreted MakA protein can bind and internalize into the HCT8 cells. Interestingly, the secreted MakA from the Δ*hapA* mutant strain could bind to the plasma membrane and internalize into the HCT8 cells (**Fig 1B and 1C**). From determination of fluorescence intensity of MakA on the surface and inside cells, respectively, we estimate that about one third of the protein was internalized (**Fig 1C**). Together, these results suggested that MakA secreted from *V*. *cholerae* could be delivered to the host epithelial cells.

### MakA binds to phosphatidic acid at the epithelial cell membrane

The cytotoxic effect of a culture supernatant from the wild type *V*. *cholerae* strain might be due to additional secreted factors and not only of HapA and MakA, since several virulence-associated factors are known to be secreted and appear in the culture supernatant, including the cholera toxin, cytolysin, and RTX toxin [22–24]. To test the specific effect(s) of the MakA protein in absence of other secreted virulence factors from *V*. *cholerae*, we used purified MakA protein [5] for further experiments. In a recent study, we tested for MakA cytotoxicity and performed RNA-sequencing analysis with HCT8 cells treated with purified MakA protein [25]. MakA caused a dose-dependent loss of cell viability and upon treatment with 250 nM and 500 nM MakA about 80% and 50%, respectively, of the HTC8 cells remained viable after 48 h [25] (**S1B Fig**). The cytotoxic effect of MakA was not only specifically observed on HCT8 cells but also on other tested colon tumor cell line (HCT116) and on a prostate cancer cell line (PC3) (**S1B Fig**).

Upon treatment of the cells with 500 nM MakA we identified statistically significant changes of expressed genes (Log2FC>1, p≤0.01) using the Metascape express analysis tools. The analysis identified enrichment of gene sets mainly involved in metabolic processes, lipid biosynthesis and cholesterol homeostasis (**Fig 1D and 1E**). The Ingenuity Pathway Analysis (IPA) further confirmed the enrichment of these gene sets (**S2A Fig**). Host lipids are attractive targets for pathogens to modulate host cell signaling and there are many indications for an important role of lipids in various stages of host–pathogen interactions [26]. Regarding the activation of genes involved in lipid biosynthesis and cholesterol homeostasis, it was recently reported that MakA mediated an increase in total cellular cholesterol in colon carcinoma cells (HCT8 cells). In addition, MakA has been shown to induce dose dependent redistribution of cholesterol to the perinuclear region of the intoxicated cell [25]. As Metascape and IPA analysis revealed activation of genes involved in cholesterol homeostasis, we decided to investigate if MakA would co-localize with cellular cholesterol. The HCT8 cells were treated with Alexa568-MakA and counterstained with filipin, and consistent with the previous study [25], MakA caused redistribution of a small pool of cholesterol to the perinuclear region of the cell. However, we observed only weak co-localization between Alexa-568MakA and filipin stained cholesterol as quantified by Pearson correlation co-efficient (**S2B Fig**). To investigate whether MakA would bind to lipids of target cell membranes, we first employed an *in vitro* protein-lipid binding assay using membrane strips coated with various lipids as described in materials and methods. Immunodetection of MakA revealed specific binding for phosphatidic acid (PA) (**Fig 1F**). The MakA binding to PA was further tested by a liposome pull-down assay. We prepared the phosphatidylcholine (PC) liposomes with or without PA and incubated them together with purified MakA protein. After incubation with MakA, the liposome preparations were separated into a pellet and a supernatant fraction by centrifugation. The liposome-bound MakA was expected to be in the pellet whereas liposome-unbound MakA would remain in the supernatant. By SDS-PAGE and immunoblot analysis using anti-MakA antiserum, we detected presence of MakA in both the pellet and supernatant of PA-containing liposomes, while in the case of PC liposomes the protein was mainly present in the supernatant fraction (**Fig 1G**). This result provided further evidence that MakA could specifically bind to PA. We also observed MakA mediated formation of oligomeric structures on the surface of PA:PC liposomes (**S1C Fig**). To quantify the interaction of MakA with PA liposomes, we performed surface plasmon resonance (SPR) analysis. MakA exhibited considerable interactions with PC:PA liposomes with the estimated K_D_ of 261.8 ± 3.2 nM (**Fig 1H**). MakA did not interact with liposomes prepared from zwitterionic 1-palmitoyl-2-oleoyl-sn-glycero-3-phosphocholine (PC) that were used as a negative control (see also below; **Fig 7D**).

**Fig 2:**
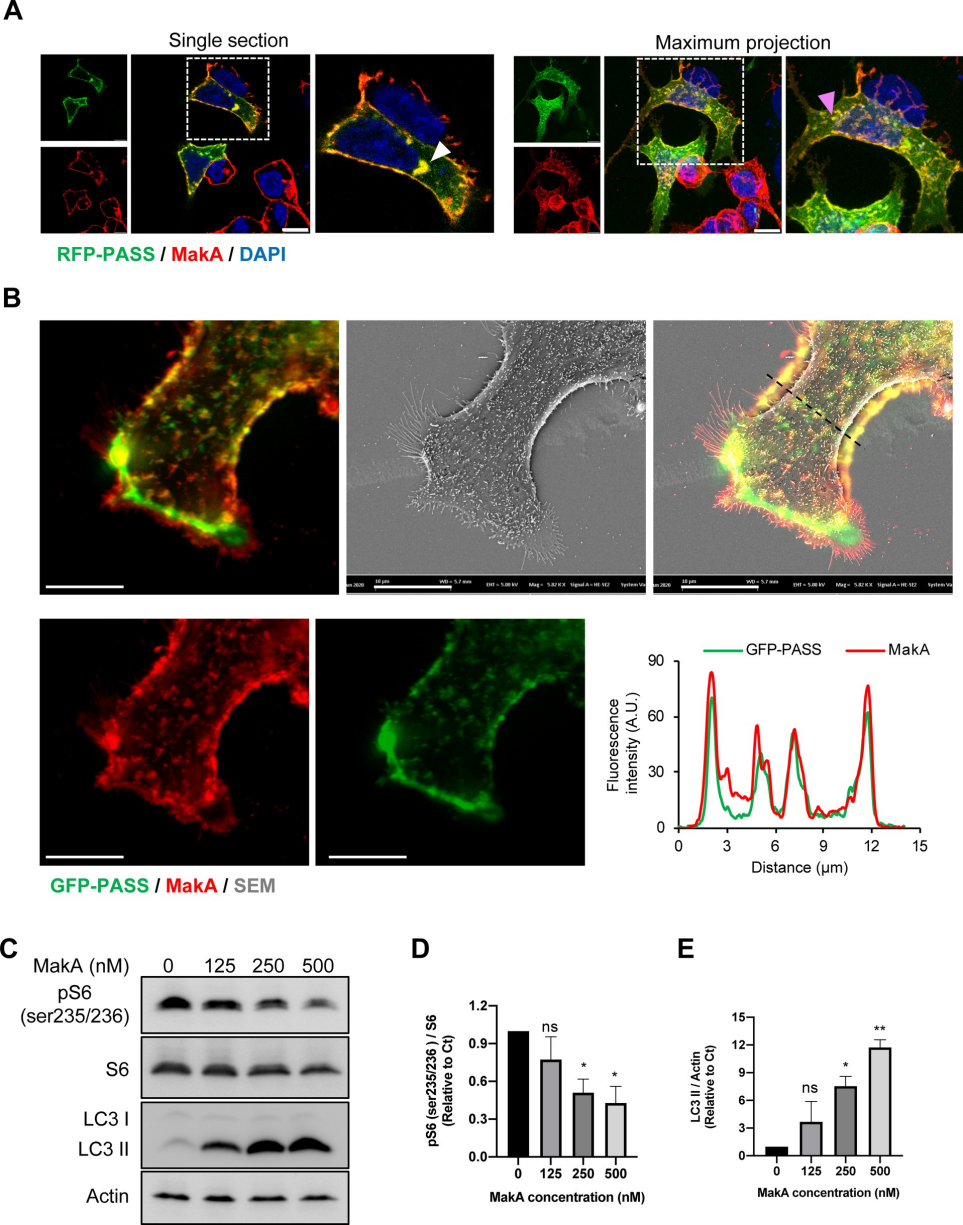
MakA co-localizes with phosphatidic acid and inhibits mTOR activity. **(A)** Cellular phosphatidic acid in HCT8 cells was visualized by overexpression of RFP-PASS (green). For co-localization experiments, HCT8 cells were treated with vehicle or MakA (250 nM) for 24 h. Cell bound MakA was detected with MakA specific antibodies (red). Cells were counterstained with DAPI (blue). Arrowheads (white) in the left panel indicates colocalization of MakA and RFP-PASS at the perinuclear aggregate of the HCT8 cells. The pictures to the right shows maximum z-stack projection of the same cells. The arrowhead (pink) indicates colocalization of MakA and RFP-PASS at the cell membrane. Scale bars, 10 μm. (**B)** Representative correlative fluorescence and scanning electron micrographs of MakA (24 h, 250 nM) treated CaCO2 cells transfected with GFP-PASS. Scale bars, 10 μm. Graph at the right bottom indicates fluorescence intensity profiles of the corresponding merge image along the dotted black line. Pearson correlation co-efficient was used for calculation of MakA (red) co-localization with GFP-PASS (green) along the filipodia like structures. (**C**) Western blot analysis of CaCO2 cells treated with increasing concentration of MakA as indicated showing levels of total S6, pS6 (ser235/236), and LC3II. Data are representative of two independent experiments. **(D-E)** Histograms indicate quantification of pS6 (ser235/236) phosphorylation normalized against total S6 or LC3 II normalized against actin. Data are representative of two independent experiments and expressed as relative to vehicle (Tris 120mM) treated control (Ct); bar graphs show mean ± s.d. Significance was determined from replicates using a one-way analysis of variance (ANOVA) with Dunnett’s post-test against vehicle control. **p<0.01, *p≤0.05. or ns = not significant. All data underlying the findings reported are shown in **S1 Data**.

To investigate if MakA would cause changes in the cellular distribution of PA, we used a RFP-tagged PA biosensor with high sensitivity (PASS) that specifically binds to cellular PA [27]. The HCT8 cells were transfected with the RFP-PASS construct and subsequently treated with MakA. By confocal microscopic analysis, we observed strong co-localization between MakA and RFP-PASS in the HCT8 cells, both at the cell membrane and at an intracellular aggregate (**Figs 2A and S3A and S3B and S3C**). To gain further insight into MakA co-localization with phosphatidic acid at host cell membranes, CaCO2 cells were transfected with GFP-PASS and treated with MakA. We used CaCO2 cells for this experiment since these cells could illustrate the morphology and many features in common with the intestinal epithelium, including microvilli [28]. To capture the spatiotemporal dynamics of PA and MakA simultaneously at the plasma membrane, a correlative light and scanning electron microscopy (CLEM) analysis was performed. A distinct co-localization was observed between MakA and GFP-PASS in filopodia-rich structures of the target cell (**Fig 2B**). The enrichment of MakA in membrane rich compartment was further confirmed by cell fractionation of MakA treated HCT8 and CaCO2 cells (**S3D and S3E Fig**). Importantly, MakA had no significant effect on total cellular PA content (**S3F Fig**). However, compared to vehicle-treated cells, MakA seemed to cause reduction of the cell membrane pool of PA (**S3G Fig**). Together these results suggested that MakA bound to PA at the filipodia-rich structures of the epithelial cell membrane.

### MakA-mediated inhibition of mTOR signaling leads to induction of autophagy in host cells

**Fig 3:**
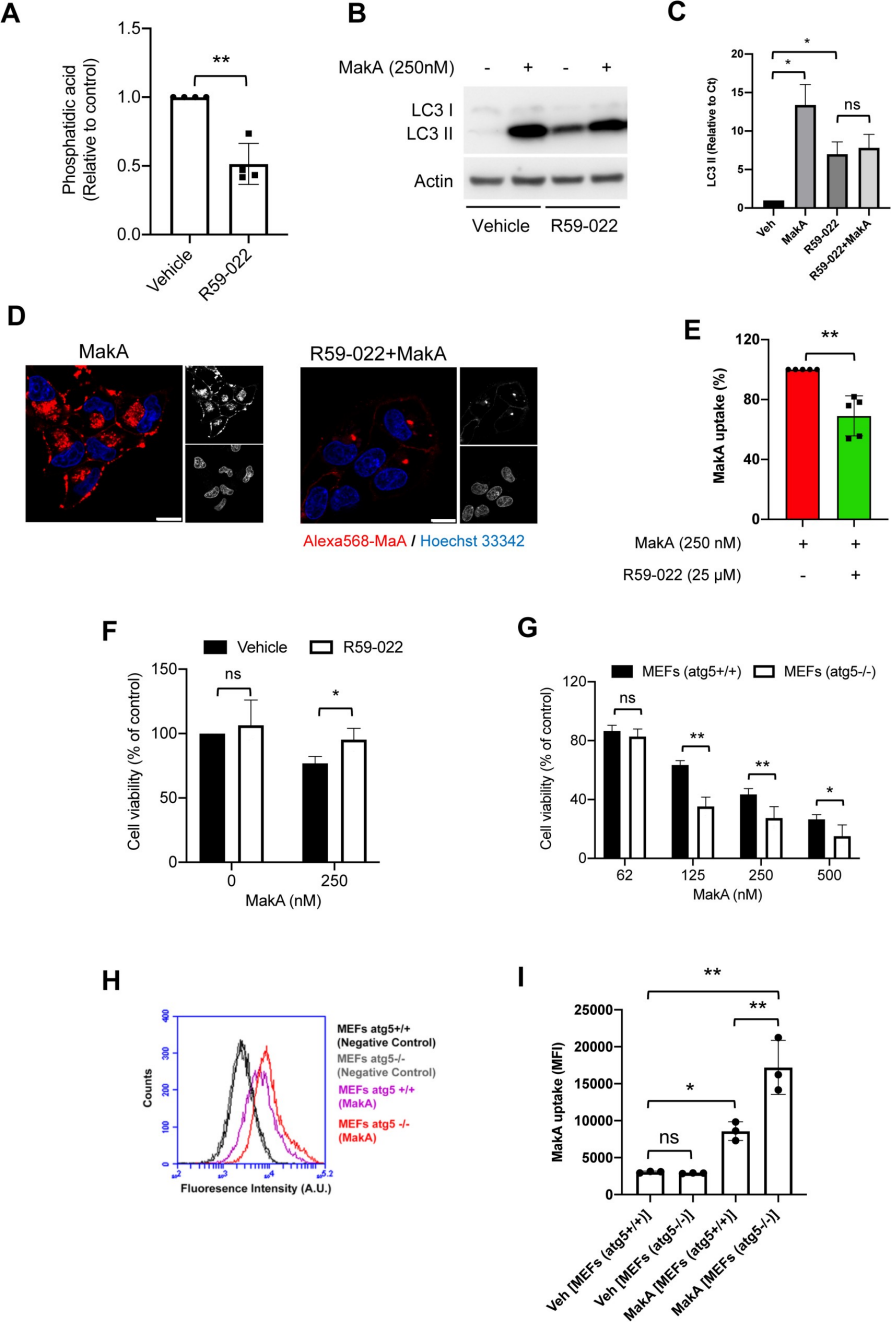
MakA inhibits phosphatidic acid mediated mTOR signaling and induces autophagy. **. (A)** Quantification of phosphatidic acid from CaCO2 cells treated with vehicle or R59-022 as indicated. Data points represent four independent experiments; bar graphs show mean ± s.d. Significance was determined from biological replicates using non-parametric t-test. **p≤0.01. (**B)** Western blot analysis of CaCO2 cells treated with MakA (250 nM) in the absence or presence of R59-022 (25 μM). (**C)** Quantification of LC3 II relative to vehicle (control) treated cells from (B). Data are representative of two independent experiments; bar graphs show mean ± s.d. Significance was determined from replicates using a one-way analysis of variance (ANOVA) with Dunnett’s post-test against vehicle control. *p≤0.05. or ns = not significant. (**D)** Confocal microscopic analysis of CaCO2 cells treated with the Alexa568-MakA (250 nM) for 24 h with or without R59-022 (25 μM). Nuclei were counterstained with Hoechst 33342. Scale bar, 10 μm. (**E)** CaCO2 cells were treated with the Alexa568-MakA (250 nM, 24 h) with or without R59-022 (25 μM); uptake was assessed by flow cytometry analysis. Bar chart represents mean fluorescence intensities (MFI) from five independent experiments; bar graphs show mean ± s.d. Significance was determined from biological replicates using non-parametric t-test. **p≤0.01. (**F)** CaCO2 cells treated with MakA (250 nM) with or without R59-022 (25 μM) for 24 h and toxicity was assessed by MTS cell viability assay. Data points represent three biologically independent experiments; bar graphs show mean ± s.d. Significance was determined from biological replicates using non-parametric t-test. *p≤0.05. (**G)** Wild type (*atg5* ^+/+^) and autophagy deficient (*atg5* ^-/-^) MEFs were treated with increasing concentrations of MakA as indicated for 48 h and toxicity was assessed by MTS cell viability assay. Data from four biologically independent experiments; bar graphs show mean ± s.d. Significance was determined from biological replicates using two way analysis of variance (ANOVA) with Sidak’s multiple comparisons test. *p≤0.05, **p≤0.01, ns = not significant. (**H)** Wild type (*atg5* ^+/+^) and autophagy deficient (*atg5* ^-/-^) MEFs were treated with 250 nM Alexa568-MakA for 24 h and uptake was assessed by flow cytometry analysis. Representative plot from three independent experiments is shown. **(I)** Histogram indicates quantification of data from (H). Data are representative of three independent experiments; bar graphs show mean ± s.d. Significance was determined from replicates using a one-way analysis of variance (ANOVA) with Sidak’s post-test. **p<0.01, *p≤0.05. or ns = not significant. All data underlying the findings reported are shown in **S1 Data**.

Membrane-bound PA has been linked to activation of several established pathways, including activation of mTOR [29,30]. In order to test the potential effect of MakA on phosphatidic acid-mediated mTOR signaling we treated HCT8 cells with increasing concentrations of MakA. Western blot analysis showed that it caused a significant decrease in mTOR activity as quantified by a concentration dependent decrease in mTOR (Ser2448) phosphorylation (**S3H and S3I Fig**). The level of total mTOR remained unchange, however (**S3H and S3J Fig**). The ribosomal S6 kinase (S6K) is the most well characterized target of mTORC1 that phosphorylates 40S ribosomal protein S6 (S6) at five serine residues (Ser235/236/240/244/247) to promote the transcription of genes involved in ribosome biogenesis. Thus, S6 phosphorylation is considered one of the most sensitive readouts of mTORC1-dependent signaling [31,32]. Our analysis showed that MakA caused a dose dependent decrease in phosphorylation of S6 (Ser235/236) (**Fig 2C and 2D**). Furthermore, the inhibition of mTOR activity led to activation of autophagy as detected by an increase in LC3 II levels (**Figs 2C and 2E and S4A and S4B**). Data from recent studies suggest that MakA actually may cause induction of non-canonical autophagy [25]. Depletion of PA has been linked to inhibition of mTOR and activation of autophagy [33]. We therefore aimed to investigate the potential role of PA in MakA induced autophagy and host cell toxicity. PA was depleted with the diacyl glycerol kinase alpha (DGK∝) inhibitor, R59-022 [34], that on its own lead to activation of autophagy as detected by induction of LC3 II (**Fig 3A and 3B and 3C**). Importantly, R59-022 decreased MakA-induced increase of LC3 II (**Fig 3B and 3C**). In addition, R59-022-mediated inhibition of PA led to decrease in MakA binding to the host cell and a decrease in MakA-induced host cell toxicity (**Fig 3D and 3E and 3F**).

**Fig 4:**
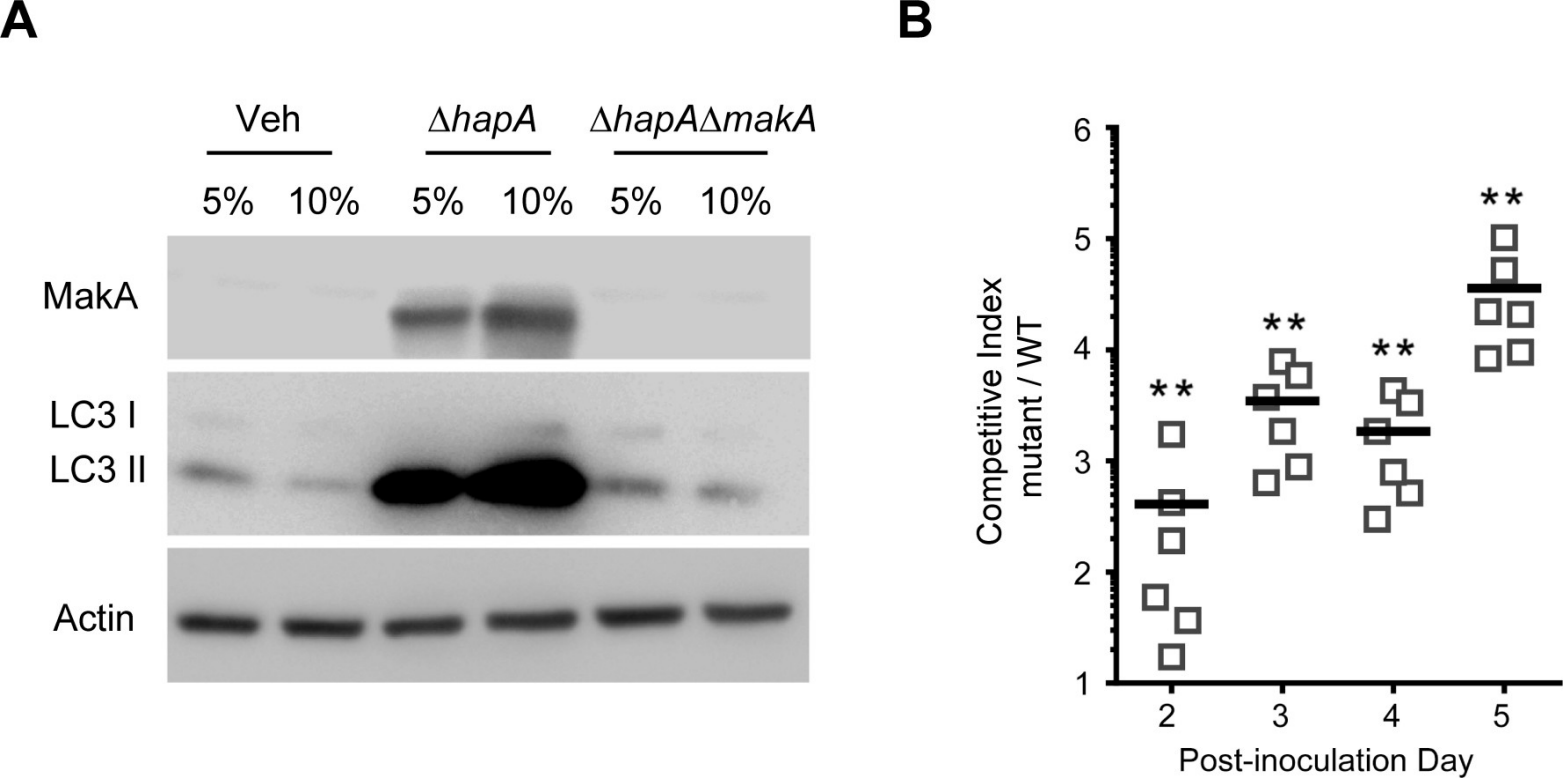
*V*. *cholerae* expresses MakA under natural infection condition and induces autophagy. **(A)** Western blot analysis of CaCO2 cells treated (24 h) with indicated concentration of supernatants collected from A1552, single mutant (Δ*hapA*) or double mutant (Δ*hapA*Δ*makA*) strain. **(B)** Five-week-old CD-1 mice were treated with streptomycin. Approximately 10^8^ cells of wild-type (*lacZ*^*-*^) and Δ*makA* mutant (*lacZ*^*+*^) were mixed in a 1:1 ratio and intragastrically administered to the adult mice. Fecal pellets were collected from each mouse and plated onto selective (for Streptomycin resistance) plates containing X-Gal to score the Lac phenotype. The competitive index was calculated as the ratio of mutant (blue, *lacZ*^*+*^) colonies to wild-type (white, *lacZ*^*-*^) colonies collected from 1g fecal material. Data was normalized to the input ratio. Horizontal line represent means from six mice. **p≤0.05. All data underlying the findings reported are shown in **S1 Data**.

To determine if autophagy would be involved in the MakA induced intoxication *per se*, HCT8 cells were treated with Alexa568-MakA for studies of potential co-localization between MakA and LC3 by confocal microscopy. The results showed that MakA induced autophagy in HCT8 cells and it co-localized with LC3 in the intracellular compartments (**S4A and S4B and S4C and S4D Fig**). The Atg5-dependent lipidation of LC3 is an important step in autophagosome formation [35]. We therefore took advantage of Atg5-deficient mouse embryonic fibroblasts (Atg5^−/−^ MEF) to investigate how the abolished autophagy activation would affect MakA-mediated cell intoxication. Treatment of wild type (Atg5^+/+^) MEF cells with MakA led to a dramatic increase in number of vacuolated cells compared to Atg5^−/−^ MEF cells (**S4E and S4F Fig**). On the other hand, Atg5^−/−^ MEF cells seemed relatively more sensitive to MakA uptake and intoxication than the Atg5^+/+^ MEF cells (**Fig 3G and 3H and 3I**). These results indicate that MakA-induced vacuolization and toxicity was partly dependent on autophagy induction. Importantly, we also observed induction of LC3 II accumulation in HCT8 cells treated with supernatants from Δ*hapA* mutant *V*. *cholerae*, whereas with the supernatants from the Δ*hapA*Δ*makA* double mutant we did not observe induction of LC3 II accumulation (**Fig 4A)**. Autophagy might act as a cellular defense pathway against MakA-induced autophagy modulation. In the previous studies, a secreted toxin from *V*. *cholerae* (VCC) was able to modulate autophagy in target cells and this autophagic response is necessary to override the cytotoxic effect of VCC and prevent cell death [36]. In our present studies, we showed that sterile culture supernatants from the *V*. *cholerae* strain A1552Δ*hapA*, as well as purified MakA, triggered autophagy.

The induction of autophagy is considered as a surveillance mechanism by the host against bacterial pathogens and its secreted toxins [37]. We therefore aimed to investigate if bacteria lacking the MakA cytotoxin, Δ*makA V*. *cholerae*, would differ in their ability to colonize the host when compared to the wild type *V*. *cholerae*. Mice were co-infected with the wild type and Δ*makA V*. *cholerae* strain A1552 in equal ratio (1:1) and their relative ability to colonize was monitored over 5 days. This *in vivo* colonization competition assay indicated that the Δ*makA* mutant *V*. *cholerae* colonized 2.5- to 4.5-fold better than the wild type (**Figs 4B and S5**). Together these results suggest that MakA plays an important role in induction of host cell autophagy and that *V*. *cholerae* lacking expression of MakA might have an advantage when colonizing its host.

### MakA protein is mainly internalized via caveolin mediated endocytosis and macropinocytosis

**Fig 5:**
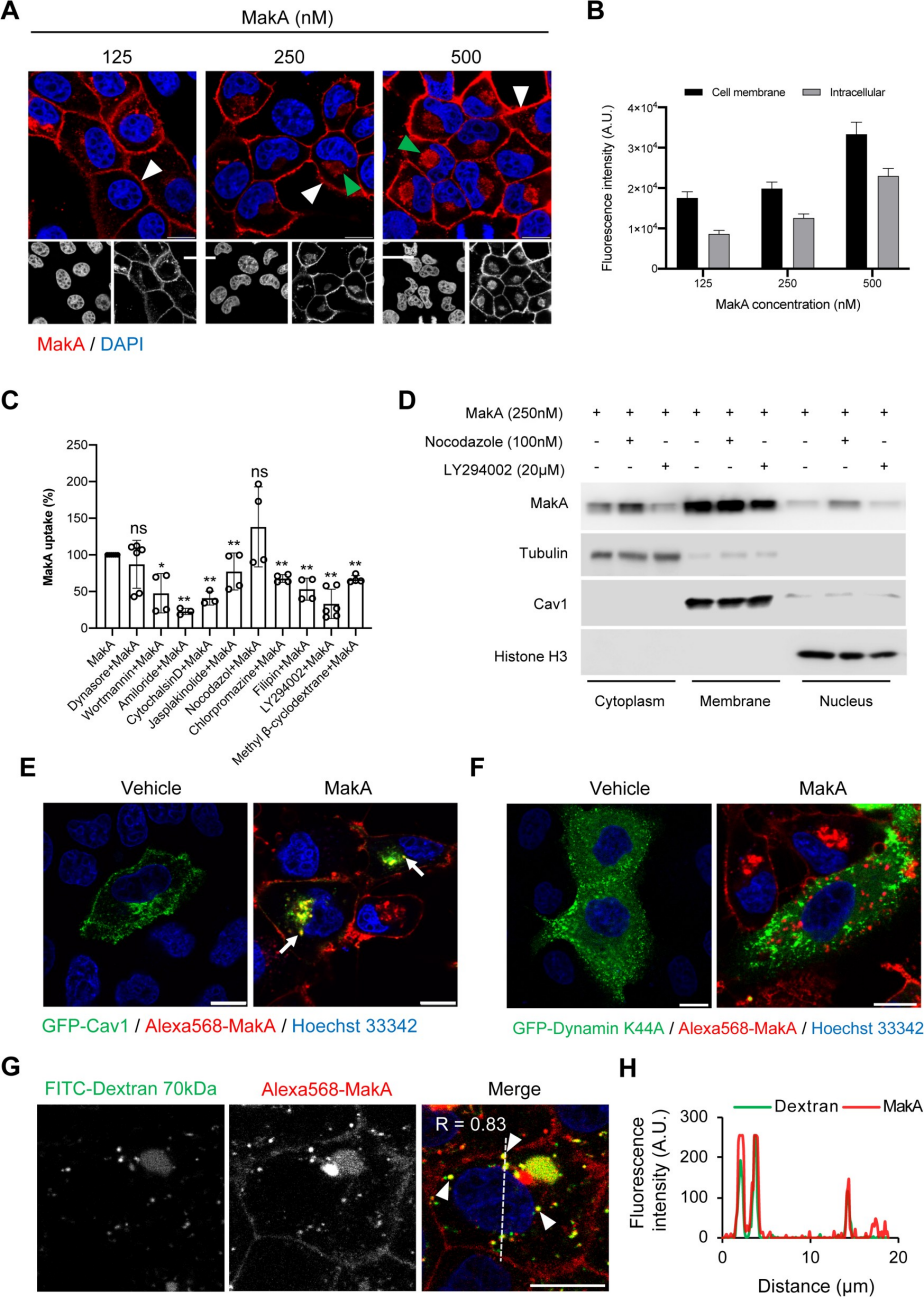
MakA protein is mainly internalized through caveolin-mediated endocytosis and micropinocytosis. **(A)** CaCO2 cells were treated with vehicle or increasing concentrations of MakA for 24 h. Cell-bound and intracellular MakA was detected with MakA-specific antibodies (red). The white arrowheads indicate cell membrane association of MakA, while the green arrowheads indicate perinuclear accumulation of MakA. Nuclei were counterstained with DAPI (blue). Scale bars, 10 μm. (**B)** Histogram indicates quantifications of cell membrane associated and intracellular uptake of MakA (n = 50 cells) for cells shown in (A). Data from two independent experiments is presented as mean ± s.e.m. **(C)** CaCO2 cells were treated with Alexa568-MakA (250 nM) for 24 h with or without endocytosis inhibitors; Dynasore (20 μM), chlorpromazine (20 μM), methyl β-cyclodextrane (2.5 mM), filipin (5 μg/mL), LY294002 (20 μM), wortmannin (5 μM), Amiloride (1 mM), cytochalasinD (500 nM), jasplakinolide (150 nM) or nocodazole (63 nM). All inhibitors were added 30 minutes prior to the addition of Alexa568-MakA. Data points represent three to six biologically independent experiments; bar graphs show mean ± s.d. Significance was determined from biological replicates using non-parametric t-test. *p≤0.05, **p≤0.01, ns = not significant. **(D)** Western blot analysis of CaCO2 cells treated with MakA (250 nM) for 24 h with or without the inhibitors; LY294002 (20 μM) or nocodazole (100 nM). The purity of different cellular fractions was confirmed by probing the membrane for Tubulin (cytoplasm), Cav1 (membrane) or Histone H3 (nuclear). Data are representative of two to three independent experiments. (**E)** CaCO2 cells transfected with GFP-Cav1 (24 h) and treated with Alexa568-MakA (250 nM, 24 h). Arrows indicate co-localization of Alexa568-MakA and GFP-Cav1 in the perinuclear region of the cell. Nuclei were stained with DAPI. Scale bar, 10 μm. (**F)** CaCO2 cells transfected with GFP-Dynamin K44A (24 h) and treated with Alexa568-MakA (250 nM) for 24 h. Nuclei were counterstained with DAPI. Scale bar, 10 μm. (**G)** CaCO2 cells treated with FITC-dextran (70kDa) (1 mg/mL) and 250 nM Alexa568-MakA for 24h. Arrowhead indicates colocalization of Alexa568-MakA and FITC-Dextran. Nuclei were counterstained with Hoechst 33342. Scale bar, 10 μm. (**H**) Fluorescence intensity profiles of the corresponding image along the dotted white line was used for calculation of Pearson correlation co-efficient. All data underlying the findings reported are shown in **S1 Data**.

We initially tested the cellular uptake of MakA in CaCO2 and DLD1 cells and observed a concentration-dependent increase in its uptake as monitored by confocal microscopy (**Figs 5A and 5B and S6A and S6B**). In addition to its presence at the cell surface, MakA also accumulated in the perinuclear region of the host cell in a concentration-dependent manner. The concentration-dependent increase in cellular uptake of MakA was further confirmed by Western blot (**S6C Fig**) and flow cytometry analyses (**S6D and S6E Fig**).

Endocytosis and post-endocytic trafficking plays a role in cell signaling, development, and host–pathogen interactions. While endocytosis primarily may have a role to traffic beneficial extracellular molecules or cellular proteins, it may also mediate entry of certain toxins, viruses, and bacteria [38]. We aimed to determine if the endocytic pathways could be involved in cellular uptake of MakA. We focused on the three major endocytic pathways that have been studied extensively, namely the i) receptor-mediated (clathrin dependent) pathway, ii) lipid raft-/caveolin-dependent pathway, and iii) macropinocytosis. For this purpose, we used a library of specific endocytic pathway inhibitors. CaCO2 cells were pretreated with individual inhibitors for 30 min and then exposed to Alexa568-MakA (250 nM) for 24 h. In order to get an unbiased overview, cellular uptake of Alexa568-MakA was quantified by flow cytometry (**Fig 5C**). Chlorpromazine, which is a specific clathrin-mediated endocytosis inhibitor [39], moderately inhibited the cellular uptake of Alexa568-MakA. In receptor-mediated endocytosis, the detachment of the budding clathrin-coated vesicle from the plasma membrane depends on the guanosine triphosphatase, dynamin [40]. Dynasore, a pharmacological inhibitor of dynamin, is known to completely block receptor-mediated endocytosis [40]. Dynasore at the tested concentration (20 μM) failed to inhibit significantly the cellular uptake of Alexa568-MakA (**Fig 5C**). Moreover, the potential contribution of caveolin-mediated endocytosis was tested by using a cholesterol-depletion molecule, methyl-β-cyclodextrin and a cholesterol-stabilizing molecule, filipin [41]. As shown in **Fig 5C**, both the inhibitors caused a rather limited inhibition of the cellular uptake of Alexa568-MakA.

We then investigated the role of macropinocytosis in cellular uptake of MakA, a process by which large volumes of extracellular fluid and dissolved molecules enter into the cell in discrete vacuoles called macropinosomes [42]. The Na+/H+ exchange inhibitor Amiloride, a classical inhibitor of macropinocytosis strongly inhibited cellular uptake of MakA (**Fig 5C**). The macropinocytosis process is known to be regulated by the phosphoinositide phosphatidylinositol 3,4,5-trisphosphate (PIP3) [43]. The conversion of phosphatidylinositol 4,5-bisphosphate (PIP2) to PIP3 by PI3 kinase (PI3K) promotes proper formation of macropinosomes and their subsequent trafficking [44]. Pharmacological inhibitors of PI3K (wortmannin and LY294002) are known inhibitors of macropinocytosis [45]. We observed that wortmannin and LY294002 significantly blocked MakA internalization when compared to MakA-treated cells without inhibitor (**Fig 5C**) suggesting that the activity of PI3K is required for the internalization of MakA.

A role for the actin cytoskeleton during clathrin-mediated endocytosis and macropinocytosis has been suggested [46,47]. We therefore examined the effect of cell-permeant compounds, cytochalasin D and jasplakinolide that perturb intracellular actin dynamics [48], and the microtubule depolymerization agent nocodazole [49] on cellular uptake of MakA. The actin cytoskeleton inhibitors moderately inhibited cellular uptake of Alexa568-MakA while nocodazole failed to do so (**Fig 5C**).

To validate the results of flow cytometry, CaCO2 cells were treated with MakA in the absence or presence of pharmacological inhibitors, LY294002 or nocodazole, and subsequently fractionated into cytoplasmic, membrane and nuclear fractions. The identity of each fraction was confirmed by respective cellular fraction marker. Consistent with flow cytometry data, LY294002 caused a detectable reduction in the level of MakA in all the three fractions, whereas with nocodazole no reduction was observed (**Figs 5D and S6F**).

Taken together, our results suggest the possible involvement of both caveolin-mediated endocytosis and macropinocytosis in case of MakA uptake. However, we need to consider that several inhibitors have been shown to have some off-target effects [45,50,51]. Therefore, in order to circumvent the potential off-target effect of the inhibitors used in the current study, we performed co-localization analysis with several endocytic pathway-specific markers. We first tested for MakA co-localization with clathrin protein. For this purpose, both CaCO2 and HCT8 cells were treated with Alexa568-MakA and subsequently stained for a clathrin marker, CLTC (**S7 Fig**). In vehicle-treated cells, clathrin showed punctate staining throughout the cytoplasm and intracellular membranes. MakA treatment caused altered clathrin distribution, leading to a perinuclear ring-like structure that seemed to encapsulate the MakA present. However, there was no significant co-localization between Alexa568-MakA and clathrin marker, CLTC (**S7 Fig**). We next investigated if co-localization occurred with caveolin and Alexa568-MakA. CaCO2 cells were initially transfected with GFP-Cav1 and subsequently exposed to Alexa568-MakA. In contrast to clathrin, caveolin appeared clearly co-localized with Alexa568-MakA (**Fig 5E and S1 Movie**). Furthermore, live cell confocal microscopy showed a dramatic change in the cellular distribution of Cav1 in response to Alexa568-MakA treatment. Most of the GFP-Cav1 was accumulated in the perinuclear region of the cell with a sudden decrease in its mobility compared to the vehicle-treated cells (**S1 Movie**).

To corroborate the results obtained using the pharmacological dynamin inhibitor dynasore, we examined the cellular uptake and localization of MakA using a dynamin dominant-negative mutant, Dyn-K44A, which previously was shown to decrease dynamin GTPase activity, thereby reducing endocytosis [52]. The cellular uptake of MakA in Dyn-K44A positive cells was limited to cytoplasmic vesicles with no evidence of perinuclear aggregate formation, indicating that activation of dynamin is required for MakA-induced perinuclear aggregate formation (**Fig 5F**). In addition, we observed clear co-localization of MakA with actin filaments stained with phalloidin (**S6G Fig**). To determine the molecular basis of the macropinocytosis-mediated endocytosis involved in intracellular uptake of MakA, we investigated the co-localization of Alexa568-MakA and FITC-dextran (70 kDa), a modern gold standard for investigating macropinocytosis [53]. Strong co-localization of Alexa568-MakA with FITC-dextran (70kDa) was detected (**Fig 5G and 5H**). Together, the data suggest that intracellular uptake of the MakA protein mainly is governed by caveolin mediated endocytosis and macropinocytosis.

### MakA binds to epithelial cells and causes disruption of intracellular organelles

**Fig 6:**
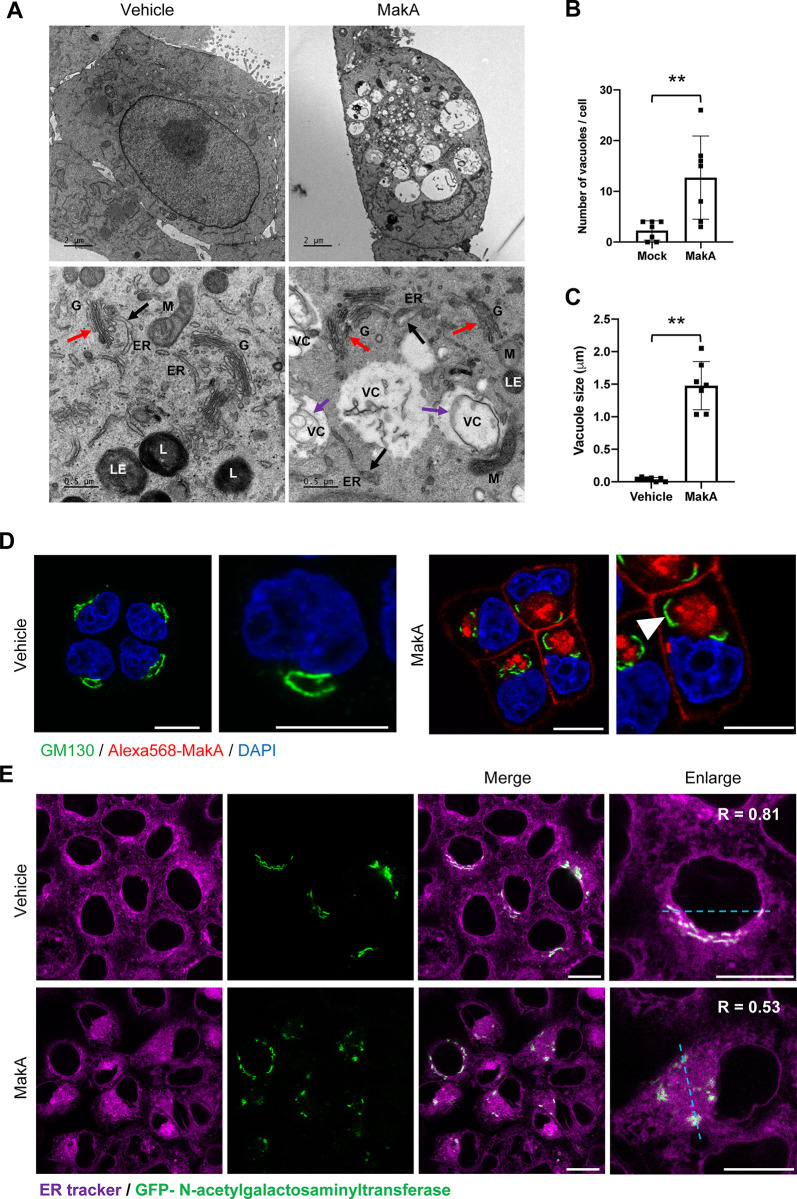
MakA causes vacuolation, disruption of endoplasmic reticulum and Golgi. **(A)** Representative electron micrographs of vehicle and MakA (250 nM, 24 h) treated CaCO2 cells. Scale bar, 2 μm. The bottom panel shows enlarged images from vehicle- and MakA-treated cells. Arrowheads indicate dilation of the endoplasmic reticulum (ER, black arrowhead), disruption of Golgi structure (G, red arrowhead) and formation of a double membrane vacuole-like structure (VC, purple arrowhead). L = Lysosomes, LE = Late endosomes and M = Mitochondria. **(B-C)** Histograms show quantification of number of vacuole **(B)** and increase in size of vacuoles **(C)** in Alexa568-MakA (250 nM, 24 h) treated CaCO2 cells**. (D)** CaCO2 cells treated with Vehicle or Alexa568-MakA (250 nM) for 24 h. Cell were stained using cis-Golgi marker, GM130. Arrowhead (white) indicates Golgi fragmentation in Alexa568-MakA treated cells. Nuclei were counterstained with DAPI. Scale bars, 10 μm. **(E)** CaCO2 cells transfected with Golgi marker, GFP-N-acetyl galactosaminyl transferase (green) and treated with Vehicle or MakA (250 nM) for 24 h. Cells were counterstained with ER-tracker (purple, 250 nM, 30 min). The dashed line was used for calculation of Pearson coefficient for colocalization. Scale bars, 10 μm. All data underlying the findings reported are shown in **S1 Data**.

To visualize the intracellular effects of MakA, CaCO2 cells were treated with MakA and imaged by transmission electron microscopy (TEM) and phase-contrast microscopy. MakA induced vacuolation in CaCO2 cells as seen by phase-contrast microscopy and TEM (**Figs 6A and S8A**). The presence of vacuoles was also observed in transmission electron micrographs of HCT8 and Cos7 cells upon treatment with MakA (**S8B and S8C Fig**). A significant increase in both number and size of MakA-induced vacuoles was observed in all cell lines tested, when compared with mock-treated cells (**Fig 6B and 6C**). The effects on detailed intracellular structures revealed: i) Golgi fragmentation, ii) dissociation of the ER-Golgi contact sites, iii) dilation of the ER membranes, iv) changes in structure of the lysosomes, and v) perinuclear accumulation of endomembrane rich aggregates. (**Fig 6A)**.

Immunofluorescence analysis confirmed disruption of the Golgi structure (**Figs 6D and S9A**). Since we observed loss of ER-Golgi contact sites in the TEM images, we investigated the co-localization of the ER and Golgi in CaCO2 cells. As expected, there was a strong co-localization (Pearson correlation co-efficient, 0.81) between the two organelles in vehicle-treated cells. In contrast, MakA treatment caused disruption of the ER-Golgi contact sites, as confirmed by loss of ER-Golgi co-localization (Pearson correlation co-efficient, 0.53), (**Fig 6E**). Additionally, Alexa568-MakA accumulated in lysosomes as shown by its co-localization with the lysosomal marker GFP-Lamp1 (**S9B Fig and S2 Movie**). Importantly, there was loss of lysosomal acidification in Alexa568-MakA treated CaCO2 cells, as observed by loss of lysotracker staining, compared to vehicle- treated cells (**S9C and S9D Fig**). Together the results suggest that membrane-binding and perinuclear accumulation of MakA lead to dysfunction of the intracellular organelles.

### Structural and functional analysis of MakA binding to phosphatidic acid

**Fig 7:**
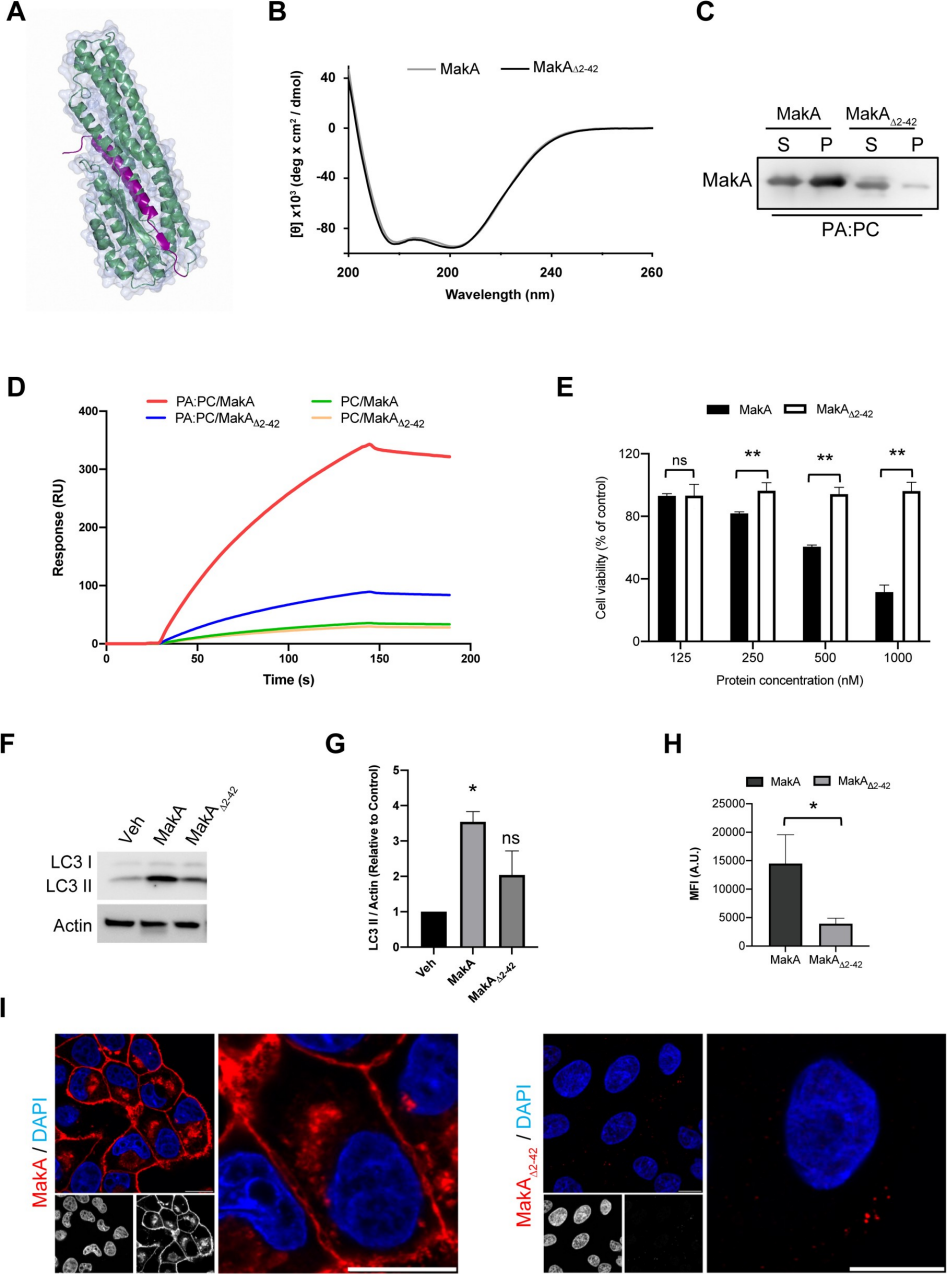
N-terminal domain of MakA plays an important role in PA binding and cellular activity. **(A)** The three-dimensional structure of MakA (PDB id: 6EZV), indicating the deleted part of the truncated variant (purple color) of MakA. The rest of the protein is depicted as a surface. (**B)** Far-UV CD spectrum of native MakA is compared with the spectra of the N-terminal (MakA_Δ2–42_) truncated variant. The mean residue ellipticity per residue [θ] (deg.cm^2^.dmol^-1^) is plotted against the wavelength. CD spectra were recorded in 20 mM phosphate buffer (pH 7.4) using 10 μM protein. Secondary structure was analyzed by CDNN version 2.1. To account for background absorption, the absorption intensity measured from control solution, containing buffer only, was subtracted. (**C)** The purified MakA native protein or the N-terminal (MakA_Δ2–42_) truncated variant (50 μg/mL) was incubated with PC or PC:PA (1:1) liposomes (20 μM) and then separated by centrifugation. Liposome-bound protein was detected using MakA specific antiserum. Data are representative of two independent experiments. **(D)** SPR analysis showing MakA (1000 nM) and MakA_Δ2–42_ (1000 nM) binding to immobilized PA:PC or PC on sensor chip L1. (**E)** CaCO2 cells were treated with the indicated concentration of native MakA or MakA_Δ2–42_ for 48 h and toxicity was assessed by MTS cell viability assay. Data points represent four biologically independent experiments; bar graphs show mean ± s.d. Significance was determined from biological replicates using two way analysis of variance (ANOVA) with Dunnett’s multiple comparisons test. **p≤0.01, ns = not significant. (**F)** Western blot analysis of CaCO2 cells treated with Vehicle (20 mM Tris), native MakA or its N-terminus deletion mutant, MakA_Δ2–42_ for 24 h. (**G)** Histogram indicates quantification of western blot from (f). Significance was determined from replicates using a one-way analysis of variance (ANOVA) with Dunnett’s post-test against vehicle control. *p≤0.05, **p≤0.01. (**H)** CaCO2 cells were treated with the Alexa568 labelled native MakA or MakA_Δ2–42_ (250 nM) for 24 h and uptake was assessed by flow cytometry analysis. Bar chart represents mean fluorescence intensities (MFI) from three independent experiments. Significance was determined from biological replicates using a two-tailed, unpaired t-test. *p≤0.05. (**I)** CaCO2 cells were treated with the native MakA or MakA_Δ2–42_ (250 nM) for 24 h and uptake was assessed by confocal microscopy using anti-MakA antiserum. Nuclei were counterstained with DAPI. Scale bars, 10 μm. All data underlying the findings reported are shown in **S1 Data**.

**Fig 8:**
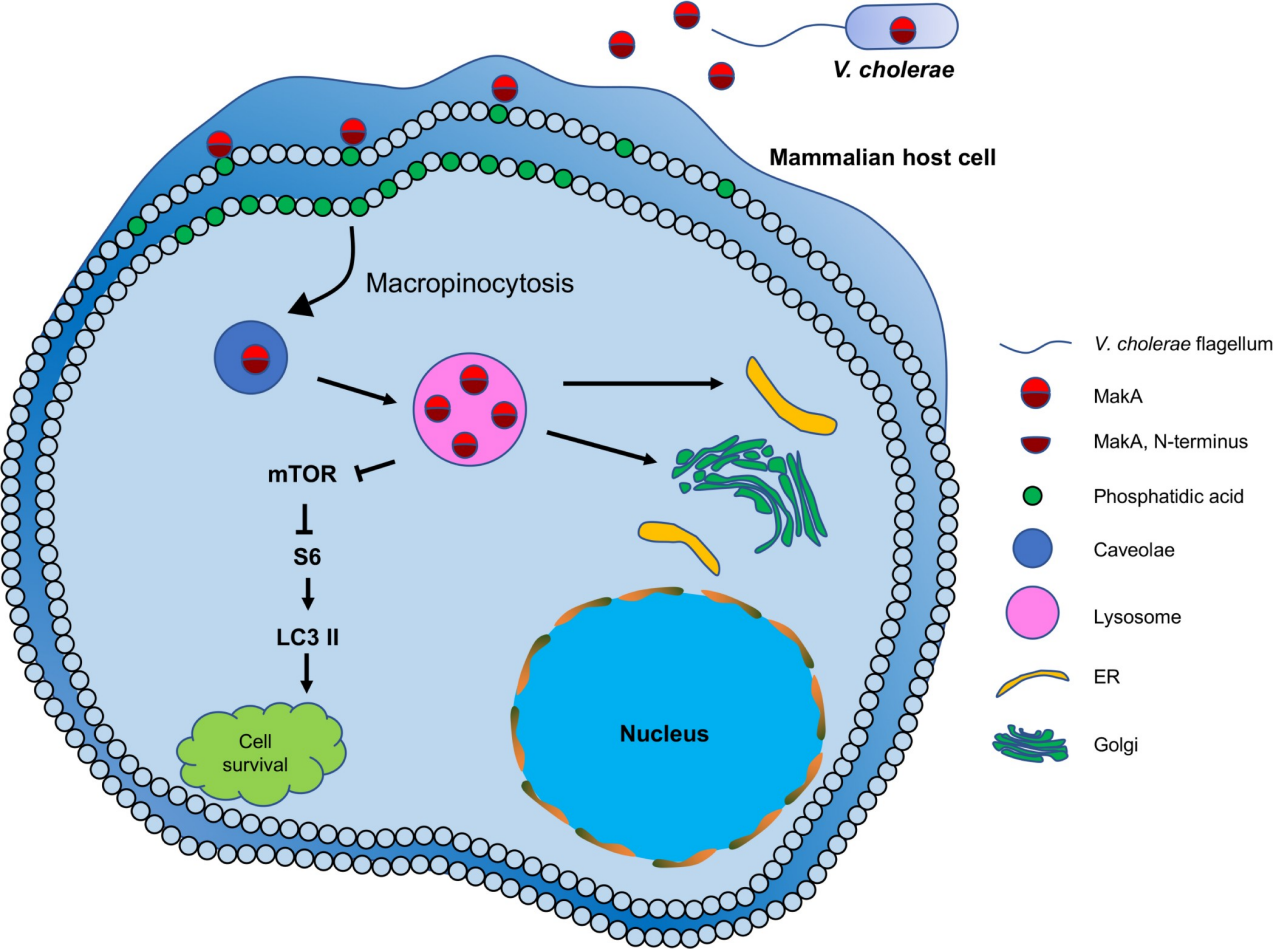
Schematic presentation of MakA binding to PA followed by its intracellular uptake. MakA secreted by *V*. *cholerae* binds to the host cell via mechanisms involving an interaction to PA in the filipodia rich region of the cell surface. The N-terminus of MakA plays a crucial role in this binding. The binding leads to macropinocytosis of the toxin to the intracellular compartments. MakA accumulates in caveolin and lysosomes. This accumulation ultimately leads to disruption of the intracellular organelles including loss of lysosomes acidification, Golgi fragmentation and ER dilation. The disruption of intracellular organelles lead to activation of autophagy that plays a protective role against MakA induced cell death.

We recently identified a potential hydrophobic (FTPP) motif at the N-terminus region (37–40 amino acid residues) of the MakA protein [5]. To examine if the N-terminus domain is essential for the cellular response to the MakA protein, we generated a variant of the MakA protein with an N-terminus deletion (MakA_Δ2–42_) (**Fig 7A**). The purity of protein was confirmed by SDS-PAGE (**S10 Fig**). The data from circular dichroism (CD) spectra indicated that the mutant protein remained structurally similar to the wild-type protein (**Fig 7B and S1 Table**). In contrast to native MakA protein, the MakA_Δ2–42_ showed weak binding for PA as evidenced by liposome pull down assay and SPR data (**Fig 7C and 7D**). Consistent with the reduced PA binding, the MakA_Δ2–42_ appeared largely inactive when tested for its ability to activate cell death or autophagy (**Fig 7E and 7F and 7G**). In addition, the MakA_Δ2–42_ protein showed weak binding to the host cell surface when compared to the native MakA protein (**Fig 7H and 7I**). Finally, we investigated the cellular distribution of MakA_Δ2–42_ and compared it to the native protein. Also the results from analysis by confocal microscopy indicated weak binding of the MakA_Δ2–42_ protein to the host cell surface and membrane, as it seemed to be present in low amounts only in intracellular cytoplasmic vesicles (**Fig 7I**). These results suggest that the N-terminal domain of MakA is essential for its binding to PA and to the host cell surface, as well as for its induction of cytotoxicity and autophagy (**Fig 8**).

## Discussion

The MakA protein was identified as a potent virulence factor of *V*. *cholerae* against *C*. *elegans* and zebrafish [5]. The present studies were aimed at identifying possible molecular interactions of the MakA protein at mammalian host cell surfaces. Our results revealed that the MakA-induced cytotoxicity and induction of autophagy included phosphatidic acid-mediated binding and internalization. Test with a mutant MakA protein suggested that the N-terminal domain was required for MakA binding to PA and thereby would be directly involved in the host cell association.

In addition to its localization on the inner leaflet of the plasma membrane of the cell, a pool of PA can also be found in the outer leaflet [54,55]. In eukaryotes, several PA-binding proteins are described including Raf-1 kinase, mTOR, and the protein-tyrosine phosphatase SHP-1 and PA is involved in the regulation of diverse cellular functions, ranging from intracellular trafficking to metabolism and cell proliferation [19,56–58]. In addition to the role of PA in regulation of other signaling cascades, PA has also been proposed to participate in controlling actin dynamics [59]. Both macropinocytosis and membrane ruffling depends profoundly on actin cytoskeleton remodeling, as evident by their complete abrogation by inhibitors that disrupt actin polymerization, like cytochalasin D. Moreover, inhibition of PI3K by either LY294002 or wortmannin has been shown to inhibit macropinocytosis [60–62]. Given that the actin-polymerization inhibitor, cytochalasin D and PI3K inhibitors, LY294002 and wortmannin blocked cellular uptake of MakA, we conclude that MakA utilizes macropinocytosis as the major endocytic pathway for its cellular uptake. These observations were further supported by inhibition of cellular entry of MakA in response to amiloride, a Na^+^/H^+^ exchangers (NHEs) blocker, that dramatically inhibits macropinocytosis [63].

Several metabolic pathways in cells lead to formation of PA. These include hydrolysis of phosphatidylcholine by phospholipase D (PLD) or phosphorylation of diacylglycerol by diacylglycerol kinases (DGK) [64]. Activation of mTOR in mammalian host cells is achieved by its binding to PA [29,30,65], that leads to autophagy inhibition. The depletion of phosphatidic acid with the DGK∝ inhibitor, R59022 has been shown to activate autophagy under certain experimental conditions [33,34]. Autophagy acts as an intracellular surveillance system to monitor and trap invading pathogens and can influence both innate and adaptive immune responses [66]. There is substantial evidence indicating the regulation of autophagy in response to both extracellular bacterial pathogens including *Streptococcus pyogenes* [67], *V*. *cholerae* [24,36] and intracellular bacterial pathogens such as *Mycobacterium tuberculosis* [68], *Shigella flexneri* [69] and *Legionella pneumophila* [70].

Little is yet known about the actual role(s) of MakA in the life cycle of *V*. *cholerae*. MakA can promote survival and proliferation in encounters with predators clearly suggests a role as fitness factor for the bacteria in natural environmental niches [25]. The findings that MakA readily interacts with human colon tumor cells and cause disruption of intracellular structures that contributed to the cytotoxic effects raise the question if and how MakA might play some role also in cholera disease. The molecular mechanism(s) involved in the disruption of ER-Golgi contact in response to MakA remain unknown. Our present observations will prompt analysis of the process in more detail in future studies. The ability of *V*. *cholerae* to colonize and multiply in the small intestine, ultimately causing the cholera disease, requires activation of a bacterial signaling cascade that leads to the production of several virulence factors. The relatively complex infection cycle of *V*. *cholerae* pathogenesis includes the repression of anti-colonization factors such as HapR [71]. During later stages of infection, when the bacterial cell density gets higher, there may be activation of HapR resulting in induction of HapA production and release from the bacterial cells. The HapA protein, an extracellular metalloprotease, promotes detachment of *V*. *cholerae* that can find a new site of infection or exit the host and initiate a new infection cycle [72,73]. Similar to HapA, MakA expression is under tight regulation of HapR [5]. Regardless of the MakA cytotoxic effect on the cultured epithelial cells, the wild type and MakA mutant *V*. *cholerae* were found to colonize in the adult mouse model with no significant difference [25]. However, in direct competition experiments with co-infection, the *makA* mutant *V*. *cholerae* showed an advantage in colonization of the adult mice over the wild type *V*. *cholerae*. We conclude from our findings that, similar to HapA, MakA may be considered as a potential anti-colonization factor of *V*. *cholerae* being expressed during later stages of cholera disease.

Several bacterial pathogens including *V*. *cholerae* during some stage of infection may activate epithelial cell exfoliation, an intrinsic host defense mechanism against the bacterial pathogens. The shedding or exfoliation of the bacterial infected host epithelial cells is thereby counteracting bacterial pathogens that replicate on the epithelium for example within the gastrointestinal tract, respiratory tract or urinary tract [74,75]. By losing the contact with the mucosa, exfoliated epithelial cells may activate autophagy and contribute as a survival mechanism of the infected tissue [76]. The components of the autophagic machinery are required for the elimination of intracellular bacteria and the secreted cytotoxic components of the extracellular bacteria [36,77]. The effector molecules from some intracellular bacteria, including *Listeria monocytogenes*, manipulate autophagy by directly interfering with the core autophagy machinery. Upon host cell infection, a pore forming toxin, Listeriolysin O (LLO) triggers a rapid inhibition of host amino acids synthesis, characterized by the inhibition of mTOR leading to induction of noncanonical autophagy involving recruitment of LC3 on the damaged Listeria-containing vacuole [78,79]. Similar to LLO, our studies revealed that MakA also caused disruption of the endolysosomal structures that may lead to inhibition of gene-sets involved in metabolism and therefore inhibition of mTOR. Results presented here suggest that autophagy induced by MakA contributed to survival of the host cells, because when autophagy was inhibited, cell death proceeded more rapidly. This observation was supported by the finding that autophagy-deficient MEFs (Atg5^−/−^) were highly sensitive to MakA-induced cell death when compared to wild-type MEFs (Atg5^+/+^).

In summary, while the MakA protein was discovered as a fitness factor that promoted survival of *V*. *cholerae* during interaction with predatory invertebrate organisms, our present findings suggest that it could potentially play a role in mammalian gastrointestinal infection. It will be of interest to uncover if the MakA activation of autophagy might cause exfoliation of epithelial cells and thereby even potentially could promote the exit and spread of the bacteria during later stages of infection.

## Materials and methods

### Ethics statement

Animal experiments were performed in accordance with the animal protocols that were approved by the Ethical Committee of Animal Experiments of Nanjing Agricultural University (permit SYXK [Su] 2017–0007).

### Bacterial strains and plasmids

All bacterial strains and plasmids used in this study are listed in **S2 Table**.

### Cell culture

CaCO2 (ATCC) and HCT8 (ATCC) cells were cultured in Roswell Park Memorial Institute (RPMI) 1640 medium (Sigma-Aldrich) supplemented with 10% fetal bovine serum (FBS), 1% penicillin/streptomycin, and non-essential amino acids at 37°C with 5% CO_2_. Cos7 (ATCC), and mouse embryonic fibroblasts (MEFs) (kind gift of Noboru Mizushima–Tokyo Medical and Dental University, Tokyo, Japan) [80] were cultured in Dulbecco’s modified Eagle medium (DMEM) (Sigma-Aldrich) supplemented with 10% fetal bovine serum (FBS), 1% penicillin/streptomycin, and non-essential amino acids at 37°C with 5% CO_2_.

### Antibodies

Anti-LC3B (#2775, WB = 1:1000, IF = 1:100), S6 (#2217, WB: 1:1000), Phospho-S6 (Ser235/236) (#2215, WB: 1:1000), mTOR (#2972, WB: 1:1000), Phospho-mTOR (Ser2448) (#2971, WB: 1:1000), antibodies were purchased from Cell Signaling Technology. Anti-MakA (GeneCust, IF = 1:100, WB = 1:15000) antisera were produced by GeneCust. Anti-beta-actin antibody (A2228, WB: 1:5000) was purchased from Sigma-Aldrich. Anti-His antibody (ab18184, WB: 1:1000) was purchased from Abcam. Goat anti-rabbit-HRP (#AS09602, WB: 1:5000) were purchased from Agrisera and Rabbit anti-mouse-HRP (#P0260, WB: 1:5000) antibodies were purchased from Dako. Alexa Fluor 488/555/647 conjugated secondary antibodies for immunofluorescence were purchased from Thermo Fisher.

### Plasmids and transfection

Cav1-GFP was a gift from Dr. Ari Helenius (Addgene plasmid # 14433; http://n2t.net/addgene:14433; RRID:Addgene_14433), GFP-Dynamin 2 K44A was a gift from Dr. Pietro De Camilli (Addgene plasmid # 22301; http://n2t.net/addgene:22301; RRID:Addgene_22301) and the phosphatidic acid-sensitive probes, GFP-PASS and RFP-PASS, were gifts from Dr. Guangwei Du. Transfection of DNA constructs was performed using polyethylenimine (PEI) mediated transient transfection according to the manufacturer’s directions (Sigma). GFP-N-acetyl galactosaminyl transferase (#C10592) and GFP-Lamp1 (#C10596) were used for transient transfection according to the manufacturer’s instructions (Thermo Fisher).

### Cloning and purification of MakA or MakA variant of *V*. *cholerae*

Cloning, overexpression and purification of wild type MakA is previously reported [5]. To generate the N-terminally truncated variant of MakA (MakA_Δ2–42_), a wild type MakA clone was used as template and the mutation was generated using the Quikchange XL-II site directed mutagenesis kit (Stratagene). The mutation was verified by DNA sequencing. Primers used for truncated variant of MakA are MakA_Δ2-42_-F 5’-gtc atc aaa cca atc tgg cat ggc gcc ctg aaa ata-3’, and MakA_Δ2-42_-R 5’-tat ttt cag ggc gcc atg cca gat tgg ttt gat gac-3’ respectively.

To express the wild type and the truncated variant of MakA, we used essentially the same protocol as reported previously [5]. Briefly, *E*. *coli* BL21(DE3) LysS (Novagen) cells expressing wild type or MakA_Δ2–42_ protein were grown until OD_600_ ~ 0.8 and expression was induced by addition of 1 mM Isopropyl β-D-1-thiogalactopyranoside (IPTG). Bacteria were harvested by centrifugation at 4^0^ C and the proteins were purified on Ni-NTA resin, followed by cleavage of the tagged N-terminal 6-histidine using TEV protease and re-purified through the Ni-NTA resin to remove the His-tag protein. The cleaved proteins were concentrated on an Amicon Ultra-15 10K molecular weight cutoff (MWCO) filter (Millipore) and further purified on a HiLoad 16/60 Superdex 200 pg gel filtration column (GE Healthcare), equilibrated with PBS pH 7.4. The purity of all proteins was confirmed by SDS-PAGE and Western blot.

AlexaFluor568 labelling of MakA and MakA_(Δ2–42)_ was performed using an Alexa Fluor568 protein labelling kit (Thermo Fisher) according to the manufacturer’s instruction.

### *V*. *cholerae* mutant construction

Mutagenesis of the *V*. *cholerae* wild-type strains A1552 and the Δ*makA* in the *hapA* locus was constructed by making in-frame deletions of the entire reading frame by using pCVD442 Δ*hapA* construct as described [5,73].

### Circular dichroism spectroscopy

Far-UV Circular Dichroism (CD) spectra of wild type MakA or the MakA_Δ2–42_ were recorded between 200–250 nm at 25°C using a Jasco J-720 Spectropolarimeter (Japan). The protein concentration used was 10 μM final in PBS, pH 7.4. The spectra were recorded using a 0.1 cm quartz cuvette, a bandwidth of 2 nm with subtracted background, and data were averaged based on five repeated scans. The secondary structures were analyzed based on neural network theory using the program CDNN version 2.1

### Correlative light and scanning electron microscopy

CaCO2 cells were grown on cover slips treated with MakA (250 nM, 24 h) in RPMI-1640 medium and fixed with paraformaldehyde (4%). MakA was detected with anti-MakA antiserum followed by incubation with secondary antibodies conjugated to Alexa-555. For co-localization of MakA with phosphatidic acid, CaCO2 cells were transfected with GFP-PASS (24 h), followed by treatment with MakA. To keep the morphology of the cells intact for the scanning electron microscopy (SEM), cells were not permeabilized during the entire staining procedure. Fluorescence images were captured as z-stack on an inverted Leica THUNDER microscope with a 63X/1.4 oil immersion objective. After fluorescence imaging the samples were coated with iridium (1 nm). In addition, the samples were dehydrated in gradient series of ethanol, starting from 70% up to 99% (2 times). For imaging, we used field-emission scanning electron microscopy (FESEM; Carl Zeiss Merlin GmbH). Image overlay of immunofluorescence images (maximum 3D projections from z-stack) and electron micrographs was performed manually using Adobe Photoshop.

### Transmission electron microscopy

CaCO2, HCT8 or Cos7 cells were plated in 6-well plates, treated with MakA (250 nM) for 48 hours in RPMI-1640 medium and fixed with paraformaldehyde (4%) and glutaraldehyde (0.5%) in 0.1M sodium cacodylate (TAAB Laboratories, Aldermaston, England) followed by another fixation in 2.5% glutaraldehyde, 0.05% Malachite green in 0.1M sodium cacodylate buffer, washed and post-fixed with 0.8% K3FC(CN)6 and 1% OsO4. Samples were further stained with 1% tannic acid and 1% uranyl acetate (EMS, Hatfield, PA), dehydrated with a graded series of ethanol and infiltrated with a graded series of Spurr’s resin (TAAB Laboratories, Aldermaston, England) with a final polymerization overnight at 60°C. All sample preparation steps were performed with the PELCO Biowave pro+ (Ted Pella, Redding, CA). 70nm sections were cut on an Ultracut UCT ultramicrotome (Leica) and collected on grids. Samples were examined in a Jeol 1230 transmission electron microscope (TEM) operating at 80kV. Micrographs were acquired with a Gatan Orius 830 2Kx2K CCD camera (Gatan, Abingdon, Great Britain) using Digital micrograph software.

For liposomes negative staining samples were added to glow discharged 300 mesh copper grids with a thin film of carbon (Ted Pella, Redding, CA) washed twice with MQ water and negatively stained with 1.5% uranyl acetate solution (EMS (Hatfield, PA). Grids were examined with Talos L120C (FEI, Eindhoven, The Netherlands) operating at 120kV. Micrographs were acquired with a Ceta 16M CCD camera (FEI, Eindhoven, The Netherlands) using TEM Image & Analysis software ver. 4.17 (FEI, Eindhoven, The Netherlands).

### Immunofluorescence

For fixed cell immunofluorescence, cells were grown in coverslip-bottom 8-well chamber slide (μ-Slide, ibidi) and fixed in 4% paraformaldehyde for 30 min at room temperature (RT), permeabilized in 0.25% Triton X-100 (15 min). Cells were blocked with 5% fetal bovine serum (60 minutes, RT) followed by a 1–2 hour incubation with primary antibody at room temperature. Cells were then washed with PBS and incubated with primary antibodies (overnight, 4°C). Subsequently, the cells were washed with PBS and incubated with respective Alexa488/555/647- conjugated secondary antibodies. For live-cell imaging, cells were seeded on coverslips at the bottom of a 8-well chamber slide (μ-Slide, ibidi) and incubated for 24 h. Imaging was performed in RPMI without phenol red (Sigma).

For detection of cellular cholesterol, we used a cell-based cholesterol assay kit (ab133116; Abcam, Cambridge, MA). Briefly, Alexa568-MakA (250 nM) or vehicle (20 mM Tris) treated HCT8 cells were fixed with PFA (3%) and stained with filipin III following the manufacturer instructions. Cells were visualized using an EZC1 Eclipse laser scanning confocal microscope (Nikon), using a 63 X /1.4 plan Apo λs lens or Leica SP8 inverted confocal system (Leica Microsystems) equipped with a HC PL APO 63x/1.40 oil immersion lens. Images were captured and processed using the NIS-Elements (Nikon) or LasX (Leica Microsystems). Fluorescence intensity profiles were generated using the plot profile command in ImageJ. Images were processed using ImageJ–FIJI distribution [81] (NIH).

### Flow cytometry

Cellular uptake of Alexa568-MakA (250 nM, 24 h) in treated CaCO2 cells, in the presence or absence of a given inhibitor as indicated in the corresponding figure legend, was investigated by flow cytometry. Live cells were gated and cellular uptake was represented as mean fluorescence intensity (MFI). For inhibitor experiments, data was either normalized to the Alexa568-MakA treated cells and expressed as a percentage, or expressed as mean fluorescence intensity (MFI). The role of autophagy in cellular uptake of Alexa568-MakA was investigated by treatment of wild type (Atg5^+/+^) or autophagy deficient (Atg5^-/-^) mouse embryonic fibroblasts (MEFs). For cellular uptake of MakA variants, wild type MEFs were treated with Alexa568 labelled MakA variants and data was expressed as mean fluorescence intensity (MFI).

### Cell viability

Cell viability of CaCO2 cells treated with MakA for the indicated time (24 h or 48 h) was quantified by MTS (Promega) or Presto Blue cell viability assays, according to manufacturer’s instructions. MTS absorbance and Presto Blue fluorescence was measured on an Infinite M200 microplate reader (Tecan). Data was normalized to the vehicle treated cells and expressed as a percentage of the control. The cell toxicity of HCT8 cells treated with bacterial supernatants was quantified by measuring propidium iodide fluorescence on an Infinite M200 microplate reader (Tecan). Data was normalized against cells treated with 0.1% triton X-100.

### Lipid overlay and liposome binding assay

The Membrane Lipid (Echelon Biosciences) was used for identification of MakA binding to lipid species. First the membranes were subjected to blocking (3% BSA prepared in 0.1% PBST, 1 h RT). After blocking, membranes were overlaid with His tag MakA (50 μg/mL, 1 h RT). Next, the membranes were incubated with an anti-His tag antibody (1:1,000 dilution, overnight at 4°C), followed by incubation with a peroxidase-conjugated goat anti-rabbit IgG antibodies (1:5000 diluted in 5% skimmed milk, 1 h). Finally, lipid-bound proteins were visualized using an ECL Western blot detection system (GE Healthcare). The Liposome binding assay was performed as previously described [82]. Briefly, PC or PA:PC (1:1), dissolved in chloroform were dried to a thin film under nitrogen stream. The lipid film was allowed to hydrate in phosphate buffer saline (PBS) or HEPES buffer saline (10 mM HEPES, 150mM NaCl, pH 7.4). This suspension was extruded over polycarbonate membranes with 0.1 μm pore size using the Avanti Mini-Extruder (Avanti Polar Lipids, Alabaster, AL, USA). The liposome suspension was diluted in three volumes of freshly prepared binding buffer (10mM HEPES, 150 mM NaCl, pH 7.0), followed by centrifugation at 21,000xg for 45 min at room temperature. After careful removal of the supernatant, the liposome pellet was resuspended in binding buffer followed by incubation with the protein of interest (5 μg/mL). To facilitate the binding, liposome-protein mixtures were incubated in the incubator for 30–60 min at 37°C. These samples were then centrifuged at 16,000xg for 30 min at room temperature. The pellets were washed in binding buffer to reduce the background. The resulting pellet was resuspended in 1X Laemmli buffer. Pelleted samples were heated at 95°C for 5 min before loading onto the SDS-PAGE. After electrophoresis the samples were transferred to nitrocellulose membrane and subjected to Western blot analysis using anti-MakA antiserum (1:1,000 dilution, overnight at 4°C) that was detected with HRP- conjugated goat anti-rabbit secondary antibodies. Images were processed using ImageJ software.

### Surface plasmon resonance

For surface plasmon resonance (SPR) experiments the sensor chip L1 (GE Healthcare Europe, Freiburg, Germany) was used to detect the lipid-MakA or MakA_Δ2–42_ interaction. All the analyses were performed at 25°C. Prior to immobilization, the flow cell surfaces were cleaned with two consecutive injections of 25 μL of 20 mM CHAPS followed by one injection of 30% ethanol at a flow rate of 30 μL/min. The PC or PA:PC (1:1) liposomes were prepared as discussed above and immobilized onto the cells of L1 sensor chip surfaces at low flow rates of 2 μL/min for 40 minutes. After lipid injection, the surface was stabilized with two 10 μl injections of 50 mM NaOH at a flow rate of 30 μL/min. The successful surface coverage was tested by injecting 40 μL of 0.1 mg/mL of Bovine Serum Albumin (BSA) at a flow rate of 5 μL/min, and a change of < 400 RU indicated sufficient coverage. Between each experiment, the L1 chip surface was cleaned by subsequent injection of 25 μL of 20 mM CHAPS, 40 mM octyl β-d-glucopyranoside and 30% ethanol, respectively at 30 μL/min. For all measurement, the flow rate was fixed at 5 μL/min. Proteins were serially diluted two fold in the SPR running buffer (10 mM HEPES, 150 mM NaCl, pH 7.0) and a volume of 40 μL with changing protein concentrations (0 to 1000 nM) were injected at flow rates of 5 μL/min. For the binding analysis with PA:PC or the PC, a maximum concentration of 1 μM each of wild-type MakA or the mutant MakA_Δ2–42_, were used. The proteins were injected for 120 sec and the dissociation was monitored for additional 120 sec. Liposomes coated on L1 chip were then regenerated by two subsequent injections of 100 mM NaOH at 30 μL/min for 1 min. The RU stability was checked, and experiments were only executed if the RU was in the range of ± 20 prior to the MakA or the mutant MakA_Δ2–42_ injection. If the RU was > 20 prior to the protein injection, the surface was regenerated with two injections each of 25 μl of 20 mM of CHAPS and 40 mM of Octyl-β-D-Glucopyranoside and fresh lipid surface was prepared for protein injection. The control flow cell was treated the same way as sample cells (liposome immobilization, stabilization, blocking with BSA, regeneration and cleaning). Experiments were repeated at least two times. Control flow cell background was subtracted from the experimental cell before further data processing. Binding affinities (K_D_) were therefore determined from concentration gradient experiments and binding responses at equilibrium were fit to a 1:1 steady-state affinity model by Scrubber 2 software (BioLogic Software Ltd., Campbell, Australia).

### Phosphatidic acid quantification

Total phosphatidic acid content of CaCO2 cells treated with increasing concentration of MakA or DGKi, R59-022 was determined using the “Total Phosphatidic Acid Assay Kit” that measures total phosphatidic acid content (PA and LPA) in cell lysate samples by a coupled enzymatic reaction system (Cell BioLabs). Briefly, cells treated with vehicle, MakA or DGKi were collected after the treatment. A small portion of the cells was used for estimation of protein concentration with the BCA assay according to the manufacturer’s instructions (Bio-Rad). The rest of the sample was processed for extraction of lipids according to the manufacturers instruction (Cell BioLabs). After lipid extraction, the lipase from the kit was used to hydrolyze the phosphatidic acid to glycerol-3-phosphate. Next, the glycerol-3-phosphate product was oxidized by glycerol-3-phosphate oxidase (GPO), producing hydrogen peroxide. The hydrogen peroxide released from this reaction reacts specifically with the kit’s Fluorometric Probe and was detected at ex. 530–560 nm and em. 585–595 nm. Total phosphatidic acid content was normalized to the total protein.

### RNA-sequencing and bioinformatics analysis

Differentially expressed genes (p-values <0.05 and log2 fold changes >1) of HCT8 cells treated with MakA (500 nM, 48 h) as previously described [25] were functionally characterized using the Metascape tools [83] and Ingenuity Pathway Analysis.

### Western blot analysis

Cells were grown on 6-well slide (3x10^5^/well, Thermo Scientific) overnight, followed by treatment with MakA for 24 h. Cells were rinsed with PBS, lysed in ice-cold lysis buffer (20 mM Tris-HCl pH 8, 300 mM KCl, 10% Glycerol, 0.25% Nonidet P-40, 0.5 mM EDTA, 0.5 mM EGTA, 1 mM PMSF, 1x complete protease inhibitor (Roche) and phosSTOP (Roche). For the extraction of different cellular compartments, Qproteome cell compartment kit (Qiagen) was used according to the manufacturer’s instruction. Cell lysates were then mixed with 4x sample buffer and boiled for 10 min prior to separation by SDS-PAGE and transferred to a nitrocellulose membrane. After a blocking (PBST, 0.1% and skim milk, 5%, RT, 1 hour), membranes were incubated with primary antibodies at 4°C (5% skim milk, overnight). After washing with PBST (0.1%) membranes were incubated with appropriate HRP-conjugated secondary antibodies in blocking buffer (5% skim milk, RT, 1 hour). Protein bands were detected with chemiluminescence reagent (Bio-Rad) using a ChemiDoc imaging system.

### Mouse colonization

The streptomycin-treated adult mouse model was used as previously described [84]. Briefly, five-week-old CD-1 mice were provided with drinking water containing 0.5% (weight/volume) streptomycin and 0.5% aspartame for 12 h before inoculation. For single colonization, approximately 10^8^ cells of wild type (Sm^R^, *lacZ*^*-*^) or Δ*makA* mutant (Sm^R^, *lacZ*^*+*^) mutant were intragastrically inoculated into each mouse. Fecal pellets were collected at the indicated time points, suspended in LB broth, serially diluted and spread on plates containing X-Gal. CFU were counted after 24 h. For the competitive colonization, a 1:1 mixture of two strains were inoculated, and the competitive index was calculated as the ratio of mutant to wild-type colonies, normalized to the input ratio. Each experiment consisted of a sample size of five to six mice.

### Statistical analysis

Data are shown as mean ± s.d. or mean ± s.e.m. Statistical significance was determined by one-way ANOVA, two-way ANOVA or by Student’s t tests (two-tailed, unpaired), as indicated in the corresponding figure legend using GraphPad Prism or Microsoft Excel. *p ≤ 0.05, **p ≤ 0.01, ns = not significant.

## Supporting information

S1 Movie Time-lapse confocal fluorescence microscopy images. Movie assembled from time-lapse confocal fluorescence microscopy images (frame rate, 4 fps; total duration of imaging, 10 min) obtained for a population of GFP-Cav1-positive CaCO2 cells. Images were acquired 24 hour after vehicle (Tris 20 mM) or Alexa568-MakA (250 nM) treatment. The vehicle-treated cells showed cytoplasmic vesicular and membrane staining of GFP-Cav1, while the Alexa568-MakA treatment induced perinuclear accumulation of GFP-Cav1 that colocalized with MakA. In addition, MakA treatment caused a decrease in mobility of the GFP-Cav1 compared to vehicle-treated cells. Nuclei were counterstained with Hoechst 33342. Scale bars, 10 μm.
(MP4)

S2 Movie Movie assembled from time-lapse confocal fluorescence microscopy images (frame rate, 4 fps; total duration of imaging, 10 min) obtained for a population of GFP-Lamp1-positive CaCO2 cells. Images were acquired 24 hours after vehicle (Tris 20 mM) or Alexa568-MakA (250 nM) treatment. The vehicle treated cells showed cytoplasmic vesicular distribution of GFP-Lamp1, while the Alexa568-MakA treatment induced perinuclear accumulation of GFP-Lamp1 that co-localized with MakA. In addition, MakA treatment caused an increase in the size of GFP-Lamp1 positive vacuoles. Nuclei were counterstained with Hoechst 33342. Scale bars, 10 μm.
(MP4)

S1 Fig MakA contributes to host cell toxicity. (A) HCT8 cells treated with supernatants (10%) from A1552, single mutant (A1552Δ*makA*), (A1552Δ*hapA*) or double mutant (Δ*hapA*Δ*makA*) strain for 24 h. Changes in cell morphology was detected by bright field microscopy. The arrowhead (black) indicates vacuolation of HCT8 cells. Red scale bars, 10 μm. (B) HCT8, PC3 and HCT116 cells were treated with increasing concentration of MakA (48 h). Loss of cell viability was measured by decrease in MTS absorbance. Mean ± s.d. of three independent experiments for HCT8 and PC3 or two independent experiment for HCT116 cells; one-way analysis of variance (ANOVA) with Dunnett’s multiple comparisons test. (*p< 0.05, **p<0.01, ns = no significant difference). (C) PA:PC liposomes were treated with vehicle (Tris 20mM) or MakA (50μg/mL) for 90 min and stained with 1.5% uranyl acetate solution. Images were captured with transmission electron microscopy (TEM). Arrowhead (blue) indicates formation of oligomeric structures present on the surface of the liposome.
(TIF)

S2 Fig Ingenuity Pathway Analysis (IPA) of MakA treated HCT8 cells. (A) Differentially expressed genes (log2FC>1 and p<0.05) of MakA treated HCT8 cells were subjected to IPA for canonical pathway analysis. Bars in brown color indicates upregulated pathways while bars in blue indicate downregulated pathways. (B) Cellular cholesterol in HCT8 cells was visualized by Filipin staining. For co-localization experiments, HCT8 cells were treated with vehicle (20mM Tris) or Alexa568-MakA (250 nM) for 24 h. Arrowhead (white) indicate perinuclear accumulation of cholesterol in response to MakA. Scale bars, 10 μm. Fluorescence intensity profiles of the corresponding image along the dotted white line was used for calculation of Pearson correlation co-efficient.
(TIF)

S3 Fig MakA co-localizes with PA and inhibits mTOR activity. (A) Cellular phosphatidic acid in HCT8 cells was visualized by overexpression of RFP-PASS (green). For co-localization experiments, HCT8 cells were treated with vehicle or MakA (250 nM) for 24 h. Cell bound MakA was detected with MakA specific antibodies (red). Cells were counterstained with DAPI (blue). Arrowheads (white) indicates co-localization of MakA and RFP-PASS. The arrowhead (green) indicates loss of cell membrane associated phosphatidic acid in response to MakA. Scale bars, 10 μm. (B) Fluorescence intensity profiles of the corresponding image in (A) along the dotted white line was used for calculation of Pearson correlation co-efficient. (C) Histogram indicates quantification of cell membrane associated and intracellular uptake of MakA (n = 50 random cells) for cells shown in (A). Data from two independent experiments is presented as mean ± s.e.m. (D-E) Subcellular fractionation (C, cytoplasm; M, membrane; N, nuclear) and Western blot analysis of HCT8 (D) and CaCO2 (E) cells after treatment with MakA (250 nM) for 24 h. The identity of different cellular fractions was confirmed by probing with antibodies specific for Tubulin (cytoplasm), Cav1 (membrane) and Histone H3 (nuclear), respectively. The histograms represent quantification of MakA from two biologically independent experiments; bar graphs show mean ± s.d. Significance was determined from biological replicates using a one-way analysis of variance (ANOVA) with Sidak’s multiple comparisons test against MakA detected in the membrane fraction of the cell. *p<0.05, **p<0.01. (F) Effect of MakA on relative level of PA in CaCO2 cells that were treated with increasing concentration of MakA. The total phosphatidic acid content was quantified with an enzymatic assay as described in Materials and Methods. The phosphatidic acid amount was normalized against the total protein. (G) Bar chart represents quantification of membrane associated phosphatidic acid from panel (A), n = 11 cells. Data points represent individual cells pooled from two independent experiments (N≥5 cells per experiment). Significance was determined from biological replicates using a two-tailed, unpaired t-test. **p<0.01. (H) Western blot analysis of CaCO2 cells treated with increasing concentration of MakA as indicated (24 h). Data are representative of two independent experiments. (I) Histogram indicate quantification of western blots from (H). Quantification of pmTOR phosphorylation normalized against total mTOR. Data are representative of three independent experiments and expressed as relative to control; bar graphs show mean ± s.d. Significance was determined from replicates using a one-way analysis of variance (ANOVA) with Dunnett’s post-test against vehicle control. *p<0.05. or ns = not significant. (J) Histogram indicate quantification of western blots from (H). Quantification of total mTOR normalized against actin. Data are representative of three independent experiments and expressed as relative to vehicle treated control; bar graphs show mean ± s.d. Significance was determined from replicates using a one-way analysis of variance (ANOVA) with Dunnett’s post-test against vehicle control. *p<0.05. or ns = not significant.
(TIF)

S4 Fig Protective role of autophagy against MakA induced cell toxicity. (A) Western blot analysis of HCT8 cells treated with increasing concentration of MakA as indicated for 24 h. (B) Histogram indicate quantification of western blots from (A). Quantification of LC3 II was normalized against actin. Data are representative of two independent experiments and expressed as relative to control; bar graphs show mean ± s.d. Significance was determined from replicates using a one-way analysis of variance (ANOVA) with Dunnett’s post-test against vehicle control. *p<0.05. or ns = not significant. (C) HCT8 cells treated with vehicle or 250 nM Alexa568-MakA (red) for 24 h. Immunofluorescence was performed using antibodies against LC3 (green). Arrowhead (white) shows co-localization of Alexa568-MakA and LC3. Nuclei were counterstained with DAPI. Scale bars, 10 μm. (D) Line graph to the right indicates fluorescence intensity profiles of the corresponding image along the dotted white line was used for calculation of Pearson correlation co-efficient. (E) Wild type (atg5^+/+^) and autophagy deficient (atg5-/-) MEFs, treated with 250 nM MakA for 24 h were imaged by phase contrast microscopy. Arrowhead (black) indicates vacuolation in wild type MEFs (atg5^+/+^) that was absent in autophagy deficient MEFs (atg5^-/-^). Scale bars, 10 μm. (F) Histogram depicts percentage of cells from 10 random fields. The number of cells in each field ranged from 15–73. Results are pooled from two independent experiments; bar graphs show mean ± s.d. Significance was determined from replicates using a one-way analysis of variance (ANOVA) with Sidak’s multiple comparisons test against vehicle control or MakA treated wild type MEFs (atg5^+/+^) and autophagy deficient MEFs (atg5^-/-^). **p<0.01. or ns = not significant.
(TIF)

S5 Fig MakA mutant V. cholerae strain has competitive advantage over the wild type. Five-week-old CD-1 mice were treated with streptomycin. Approximately 10^8^ cells of wild-type (*lacZ-*) and Δ*makA* mutant (*lacZ^+^*) were mixed in a 1:1 ratio and intragastrically administered to the adult mice. Fecal pellets were collected from each mouse and plated onto selective (for Streptomycin resistance) plates containing X-Gal to score the Lac phenotype. The competitive index was calculated as the ratio of mutant (blue, *lacZ^+^*) colonies to wild-type (white, *lacZ^-^*) colonies collected from 1g fecal material (FM) or small intestine (SI) homogenate on day 5 of the experiment. Data was normalized to the input ratio. Horizontal line represent means from five to six mice.
(TIF)

S6 Fig Cellular uptake of MakA to the epithelial cells is concentration dependent. (A) DLD1 cells were treated with vehicle or increasing concentrations of MakA for 24 h. Cell-bound and intracellular MakA was detected with MakA-specific antibodies (red). The white arrowheads indicate cell membrane association of MakA, while the green arrowheads indicate perinuclear accumulation of MakA. Nuclei were counterstained with DAPI (blue). Scale bars, 10 μm. (B) Histogram indicates quantifications of cell membrane associated and intracellular uptake of MakA (n = 50 cells) for cells shown in (A). Data is presented as mean ± s.e.m. (C) Western blot analysis of CaCO2 cells treated with increasing concentrations of MakA (24 h). Data are representative of two independent experiments. The numbers below indicates quantification of MakA relative to actin. (D-E) CaCO2 cells were treated with increasing concentrations of Alexa568-MakA (24 h). Flow cytometry analysis indicates concentration dependent increase in cellular uptake of Alexa568-MakA. Data points represents five biologically independent experiments; bar graphs show mean ± s.d. Significance was determined from biological replicates using a one-way analysis of variance (ANOVA) with Dunnett’s post-test against vehicle control. *p<0.05, **p<0.01. (F) Quantification of Western blot of CaCO2 cells treated with MakA (250 nM) for 24 h with or without the inhibitors; LY294002 (20 μM) or nocodazole (100 nM). The histogram represents quantification of MakA from three biologically independent experiments; bar graphs show mean ± s.d. Significance was determined from biological replicates using a one-way analysis of variance (ANOVA) with Sidak’s multiple comparisons test against MakA detected in the membrane fraction of the cell. *p<0.05, **p<0.01. (G) HCT8 cells were treated with MakA (250 nM) for 24 h. Cellular localization of MakA was detected using anti-MakA antiserum, while actin filaments were stained with FITC-labelled Phalloidin. Arrowheads indicate colocalization of MakA (red) and actin filaments (green). Nuclei were stained with DAPI. Scale bar, 10 μm.
(TIF)

S7 Fig Clathrin and actin accumulates around the perinuclear aggregate of MakA. CaCO2 or HCT8 cells treated with Alexa568-MakA (250 nM) for 24 h. Clathrin was detected using anti-clathrin heavy chain antibody (CLTC). Arrowhead indicates accumulation of CLTC (green) around the perinuclear aggregate of Alexa568-MakA (red). Nuclei were counterstained with DAPI. Scale bar, 10 μm.
(TIF)

S8 Fig MakA causes vacuolation in various cell types. (A) CaCO2 cells treated with MakA (250 nM) for 24 h were visualized by phase contrast microscopy. Arrowhead (red) indicates vacuolation of the CaCO2 cells in response to MakA. (B-C) Representative electron micrographs of vehicle and MakA (24 h, 250 nM) treated HCT8 and Cos7 cells. Scale bar, 5 μm. Arrowhead (red) in the right panel indicates the presence of membranes inside the vacuoles of HCT8 and Cos7 cells. Scale bar, 1 μm.
(TIF)

S9 Fig MakA causes disruption of intracellular organelles. (A) CaCO2 cells transfected with Cell Light Golgi-GFP were treated with vehicle or 250 nM Alexa568-MakA for 24h. Arrowhead (white) indicates Golgi fragmentation in Alexa568-MakA treated cells. Nuclei were counterstained with Hoechst 33342. Scale bars, 10 μm. (B) CaCO2 cells transfected with Cell Light Lysosomes-GFP (24 h) and treated with Vehicle (Tris 20 mM) or Alexa568MakA (250 nM) for 24h. Arrowhead (white) indicate lysosomes stained positive for GFP-Lamp1 and Alexa568MakA, while arrowhead (gray) indicates enlargement of lysosomes. Nuclei were counterstained with Hoechst 33342. Scale bars, 10 μm. (C) CaCO2 cells treated with vehicle or Alexa568-MakA (red, 250 nM) for 24h. Cells were counterstained with Lysotracker (green, 500 nM, 30 min). Nuclei were counterstained with Hoechst 33342. Arrowhead indicates loss of lysotracker staining in MakA positive cells. Scale bars, 10 μm. (D) HCT8 cells were treated with increasing concentrations of MakA (24 h) and stained with Lysotracker (500nM, 30 min). Flow cytometry analysis indicates concentration dependent decrease in lysotracker staining. Data points represents two to three biologically independent experiments; bar graphs show mean ± s.d. Significance was determined from biological replicates using a one-way analysis of variance (ANOVA) with Sidak’s multiple comparisons test against Vehicle (Veh) treated cell. **p<0.01.
(TIF)

S10 Fig SDS-PAGE for purity of MakA and MakA_Δ2–42_. The MakA and MakA_Δ2–42_ proteins were purified as described in the materials and methods. The protein samples were run on a SDS-PAGE and the gel was stained with Coomassie blue stain.
(TIF)

S1 Table Conformational analysis of wild-type MakA and the MakA truncated variant, MakA_Δ2–42_ for structural integrity by CD analysis. (DOCX)

S2 Table Bacterial strains and plasmids used in this study. (DOCX)

S1 Data Data underlying the findings reported. (XLSX)
