# Supplementary figures and images for: Phosphatidic acid-mediated binding and mammalian cell internalization of the Vibrio cholerae cytotoxin MakA
Source: PLoS Pathog. 2021 Mar 18;17(3):e1009414. doi: 10.1371/journal.ppat.1009414 (PMC8009392; doi:10.1371/journal.ppat.1009414)

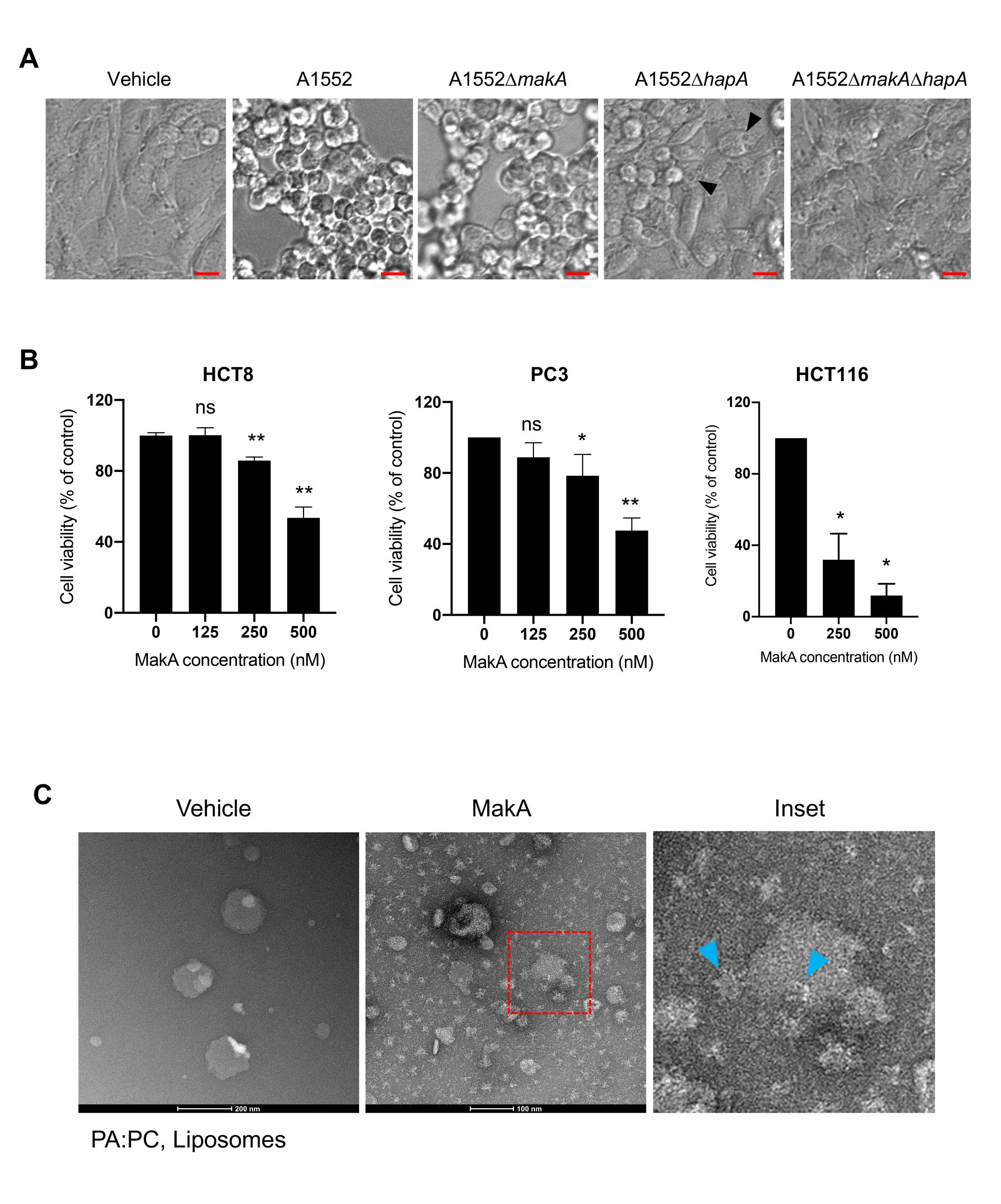

Supplement: S1 Fig — (A) HCT8 cells treated with supernatants (10%) from A1552, single mutant (A1552ΔmakA), (A1552ΔhapA) or double mutant (ΔhapAΔmakA) strain for 24 h. Changes in cell morphology was detected by bright field microscopy. The arrowhead (black) indicates vacuolation of HCT8 cells. Red scale bars, 10 μm. (B) HCT8, PC3 and HCT116 cells were treated with increasing concentration of MakA (48 h). Loss of cell viability was measured by decrease in MTS absorbance. Mean ± s.d. of three independent experiments for HCT8 and PC3 or two independent experiment for HCT116 cells; one-way analysis of variance (ANOVA) with Dunnett’s multiple comparisons test. (*p< 0.05, **p<0.01, ns = no significant difference). (C) PA:PC liposomes were treated with vehicle (Tris 20mM) or MakA (50μg/mL) for 90 min and stained with 1.5% uranyl acetate solution. Images were captured with transmission electron microscopy (TEM). Arrowhead (blue) indicates formation of oligomeric structures present on the surface of the liposome. (TIF) [file ppat.1009414.s003.tif]

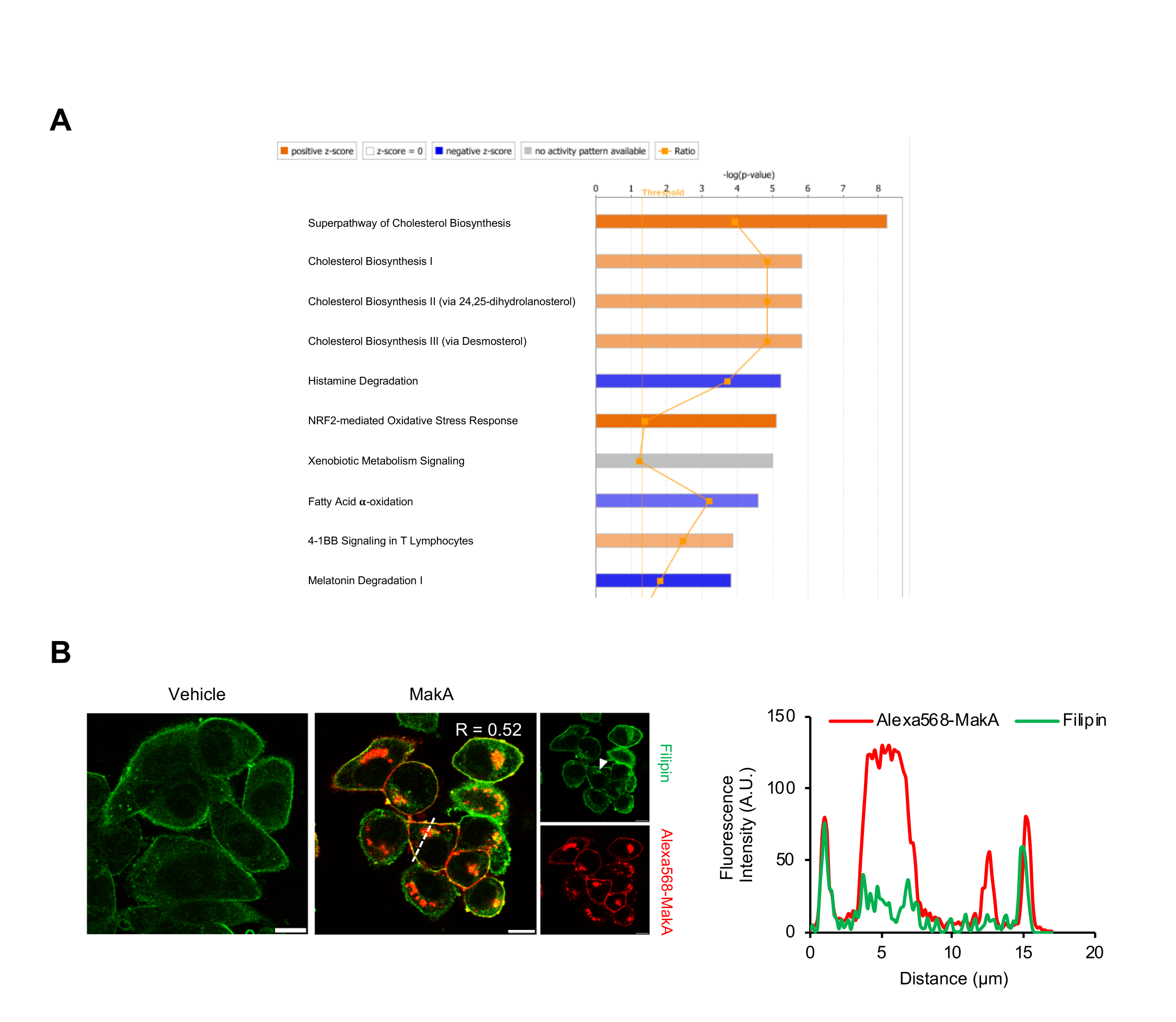

Supplement: S2 Fig — (A) Differentially expressed genes (log2FC>1 and p<0.05) of MakA treated HCT8 cells were subjected to IPA for canonical pathway analysis. Bars in brown color indicates upregulated pathways while bars in blue indicate downregulated pathways. (B) Cellular cholesterol in HCT8 cells was visualized by Filipin staining. For co-localization experiments, HCT8 cells were treated with vehicle (20mM Tris) or Alexa568-MakA (250 nM) for 24 h. Arrowhead (white) indicate perinuclear accumulation of cholesterol in response to MakA. Scale bars, 10 μm. Fluorescence intensity profiles of the corresponding image along the dotted white line was used for calculation of Pearson correlation co-efficient. (TIF) [file ppat.1009414.s004.tif]

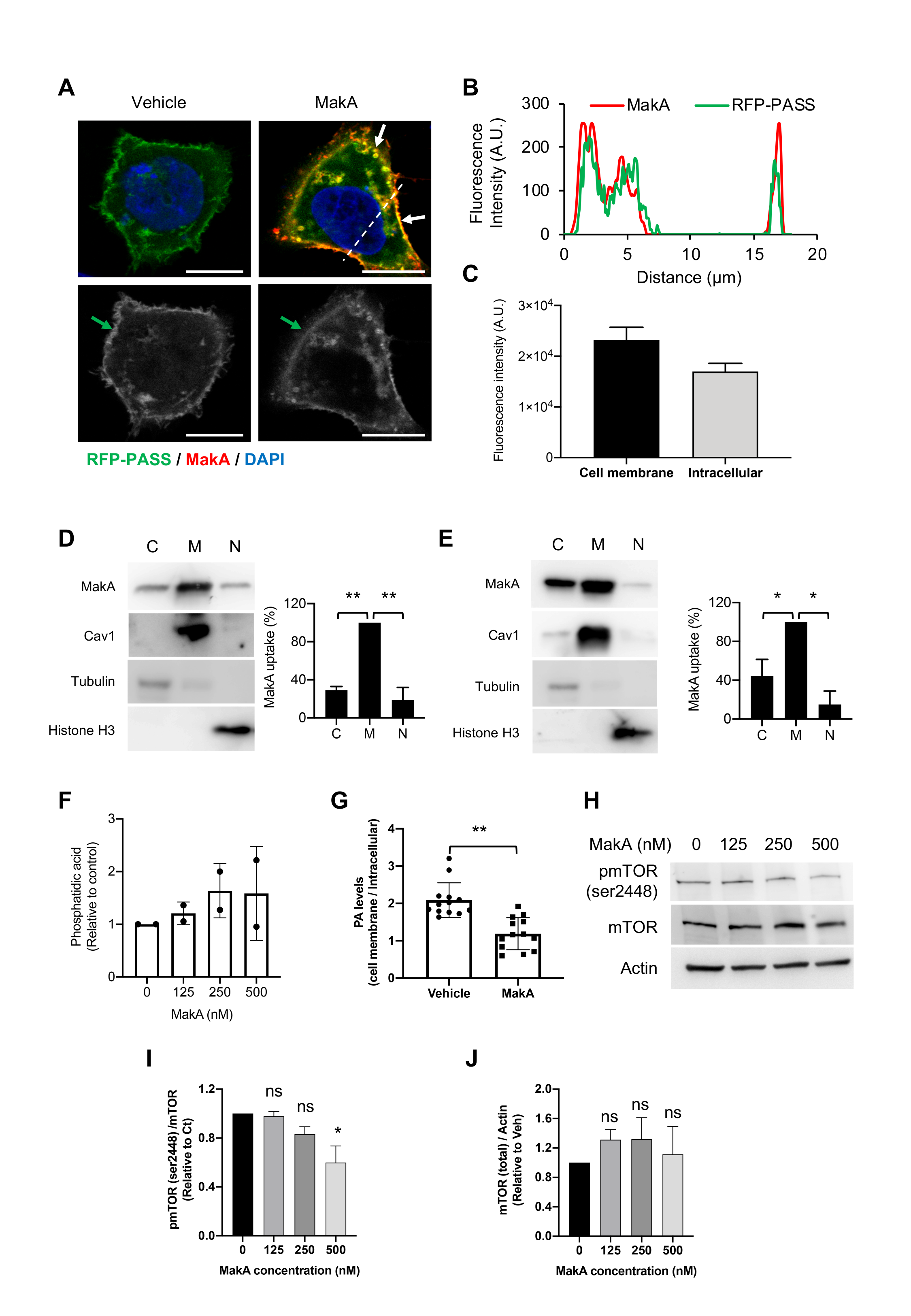

Supplement: S3 Fig — (A) Cellular phosphatidic acid in HCT8 cells was visualized by overexpression of RFP-PASS (green). For co-localization experiments, HCT8 cells were treated with vehicle or MakA (250 nM) for 24 h. Cell bound MakA was detected with MakA specific antibodies (red). Cells were counterstained with DAPI (blue). Arrowheads (white) indicates co-localization of MakA and RFP-PASS. The arrowhead (green) indicates loss of cell membrane associated phosphatidic acid in response to MakA. Scale bars, 10 μm. (B) Fluorescence intensity profiles of the corresponding image in (A) along the dotted white line was used for calculation of Pearson correlation co-efficient. (C) Histogram indicates quantification of cell membrane associated and intracellular uptake of MakA (n = 50 random cells) for cells shown in (A). Data from two independent experiments is presented as mean ± s.e.m. (D-E) Subcellular fractionation (C, cytoplasm; M, membrane; N, nuclear) and Western blot analysis of HCT8 (D) and CaCO2 (E) cells after treatment with MakA (250 nM) for 24 h. The identity of different cellular fractions was confirmed by probing with antibodies specific for Tubulin (cytoplasm), Cav1 (membrane) and Histone H3 (nuclear), respectively. The histograms represent quantification of MakA from two biologically independent experiments; bar graphs show mean ± s.d. Significance was determined from biological replicates using a one-way analysis of variance (ANOVA) with Sidak’s multiple comparisons test against MakA detected in the membrane fraction of the cell. *p<0.05, **p<0.01. (F) Effect of MakA on relative level of PA in CaCO2 cells that were treated with increasing concentration of MakA. The total phosphatidic acid content was quantified with an enzymatic assay as described in Materials and Methods. The phosphatidic acid amount was normalized against the total protein. (G) Bar chart represents quantification of membrane associated phosphatidic acid from panel (A), n = 11 cells. Data points represent indiv [file ppat.1009414.s005.tif]

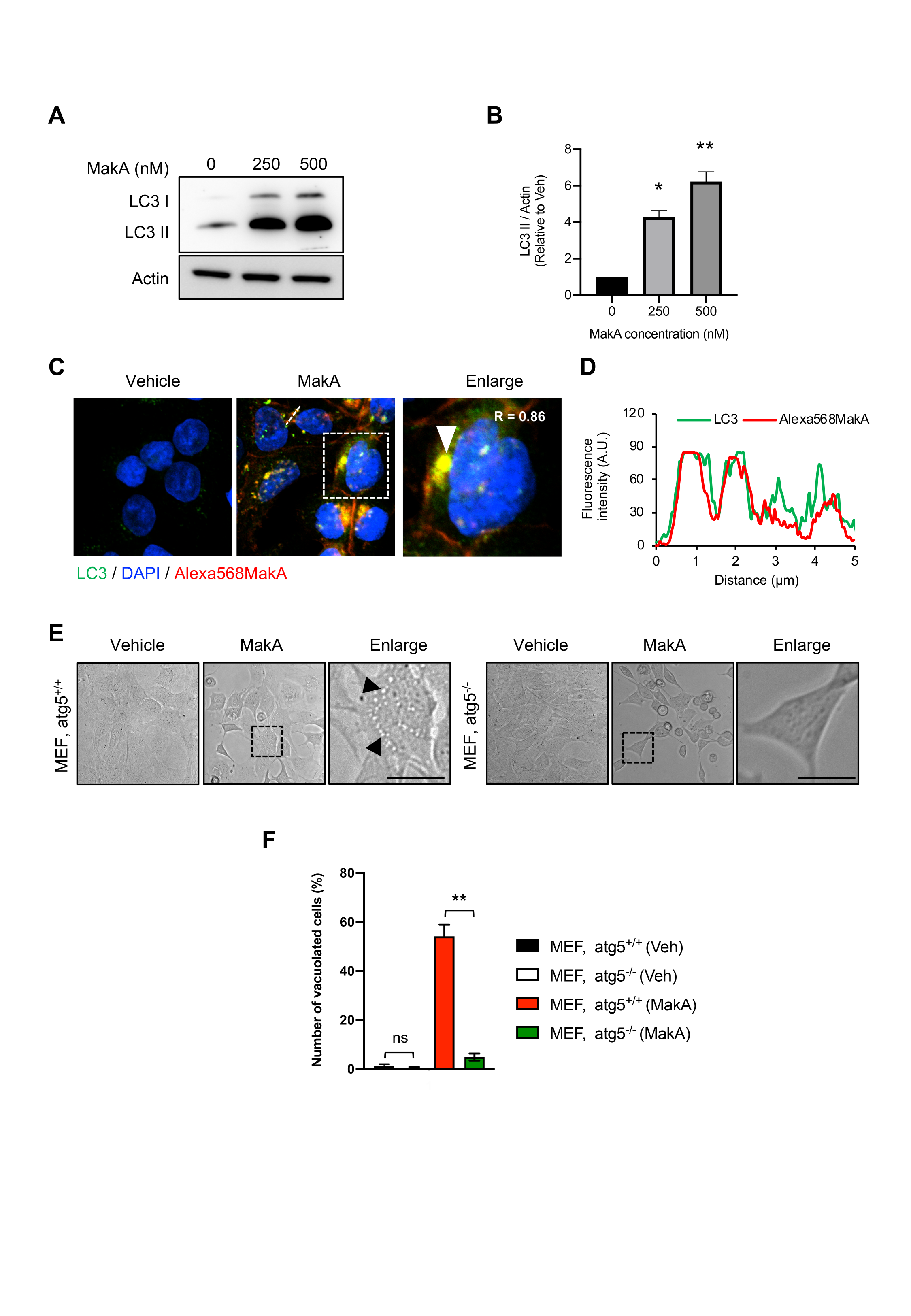

Supplement: S4 Fig — (A) Western blot analysis of HCT8 cells treated with increasing concentration of MakA as indicated for 24 h. (B) Histogram indicate quantification of western blots from (A). Quantification of LC3 II was normalized against actin. Data are representative of two independent experiments and expressed as relative to control; bar graphs show mean ± s.d. Significance was determined from replicates using a one-way analysis of variance (ANOVA) with Dunnett’s post-test against vehicle control. *p<0.05. or ns = not significant. (C) HCT8 cells treated with vehicle or 250 nM Alexa568-MakA (red) for 24 h. Immunofluorescence was performed using antibodies against LC3 (green). Arrowhead (white) shows co-localization of Alexa568-MakA and LC3. Nuclei were counterstained with DAPI. Scale bars, 10 μm. (D) Line graph to the right indicates fluorescence intensity profiles of the corresponding image along the dotted white line was used for calculation of Pearson correlation co-efficient. (E) Wild type (atg5+/+) and autophagy deficient (atg5-/-) MEFs, treated with 250 nM MakA for 24 h were imaged by phase contrast microscopy. Arrowhead (black) indicates vacuolation in wild type MEFs (atg5+/+) that was absent in autophagy deficient MEFs (atg5-/-). Scale bars, 10 μm. (F) Histogram depicts percentage of cells from 10 random fields. The number of cells in each field ranged from 15–73. Results are pooled from two independent experiments; bar graphs show mean ± s.d. Significance was determined from replicates using a one-way analysis of variance (ANOVA) with Sidak’s multiple comparisons test against vehicle control or MakA treated wild type MEFs (atg5+/+) and autophagy deficient MEFs (atg5-/-). **p<0.01. or ns = not significant. (TIF) [file ppat.1009414.s006.tif]

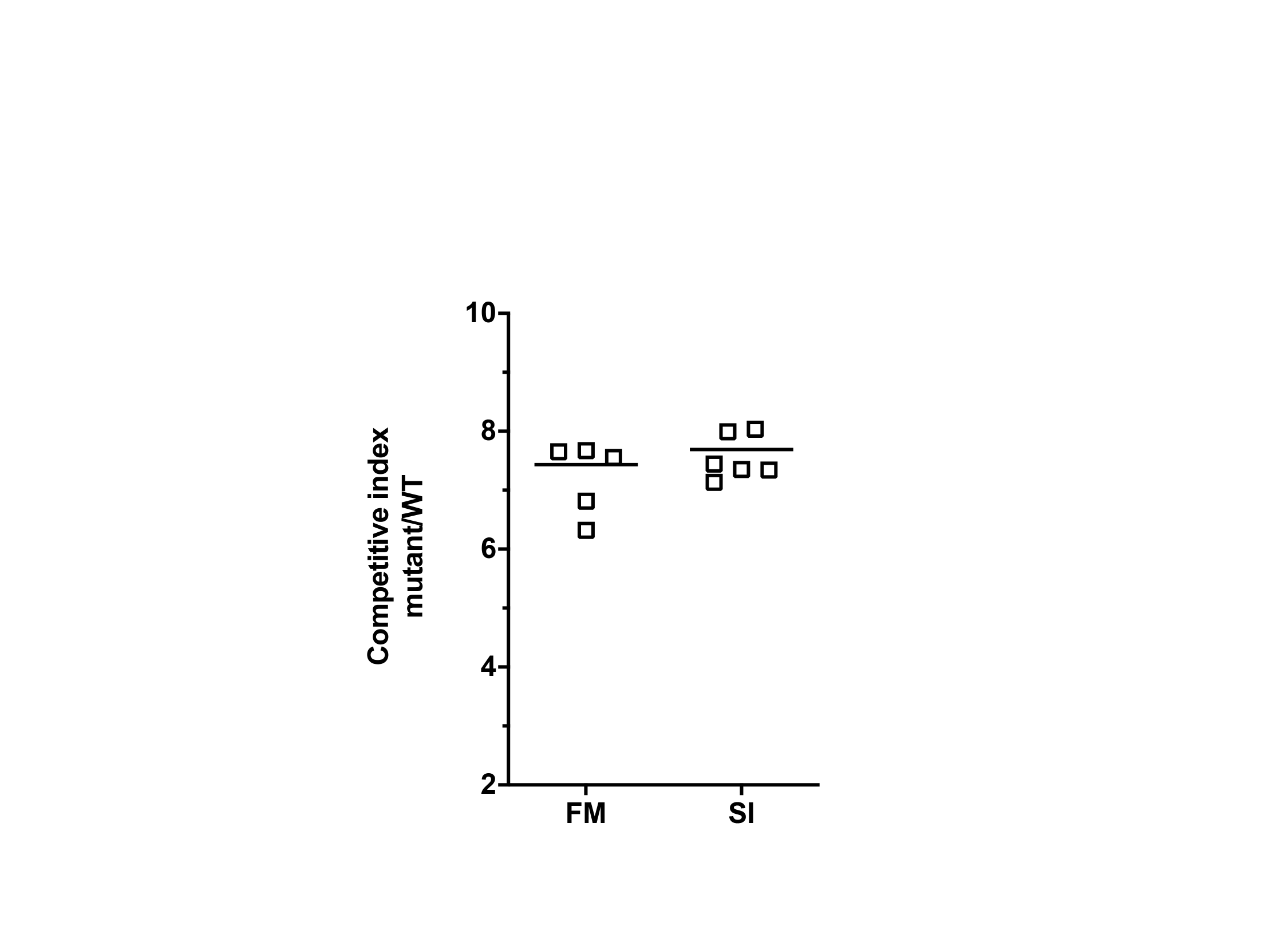

Supplement: S5 Fig — Five-week-old CD-1 mice were treated with streptomycin. Approximately 108 cells of wild-type (lacZ-) and ΔmakA mutant (lacZ+) were mixed in a 1:1 ratio and intragastrically administered to the adult mice. Fecal pellets were collected from each mouse and plated onto selective (for Streptomycin resistance) plates containing X-Gal to score the Lac phenotype. The competitive index was calculated as the ratio of mutant (blue, lacZ+) colonies to wild-type (white, lacZ-) colonies collected from 1g fecal material (FM) or small intestine (SI) homogenate on day 5 of the experiment. Data was normalized to the input ratio. Horizontal line represent means from five to six mice. (TIF) [file ppat.1009414.s007.tif]

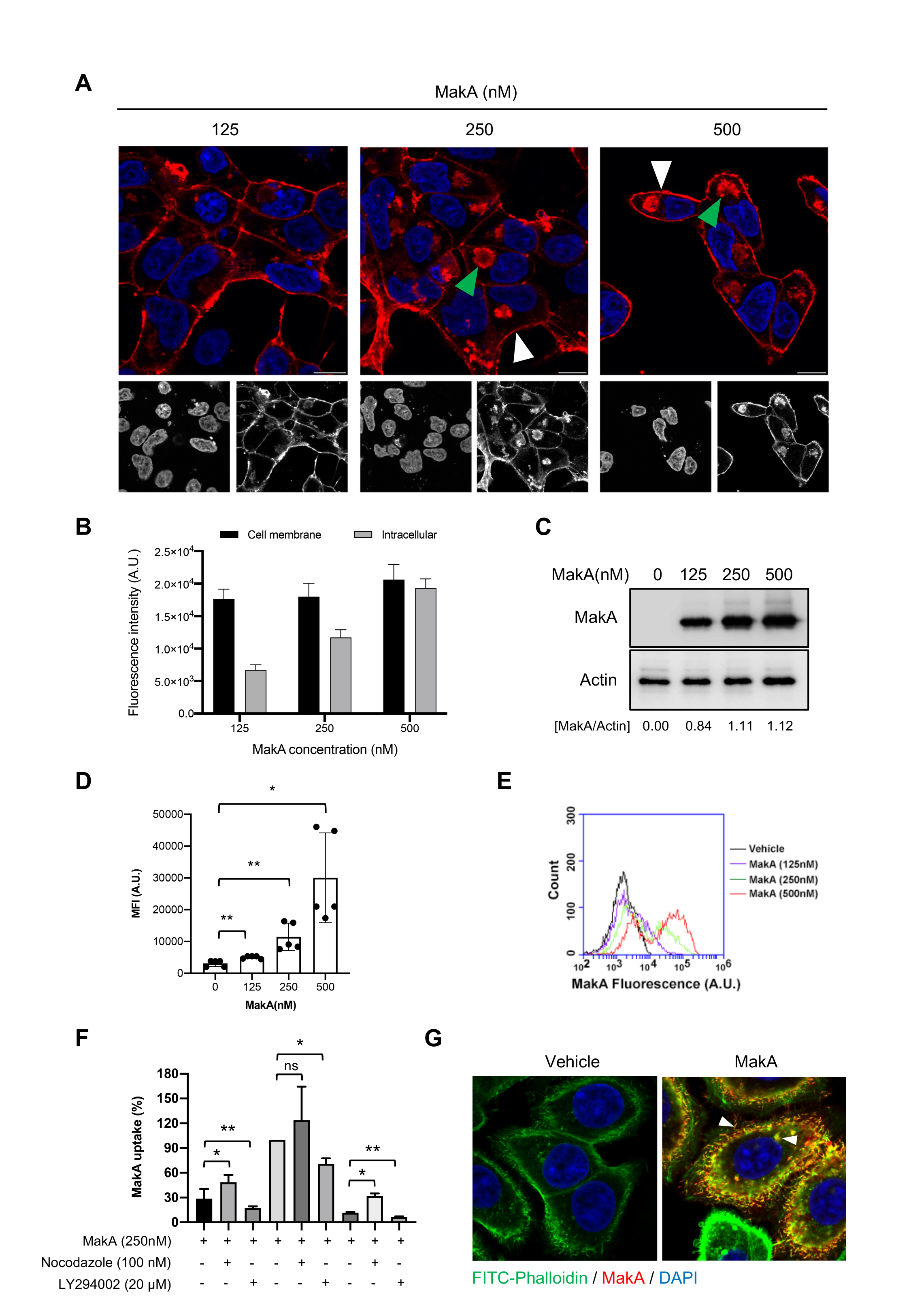

Supplement: S6 Fig — (A) DLD1 cells were treated with vehicle or increasing concentrations of MakA for 24 h. Cell-bound and intracellular MakA was detected with MakA-specific antibodies (red). The white arrowheads indicate cell membrane association of MakA, while the green arrowheads indicate perinuclear accumulation of MakA. Nuclei were counterstained with DAPI (blue). Scale bars, 10 μm. (B) Histogram indicates quantifications of cell membrane associated and intracellular uptake of MakA (n = 50 cells) for cells shown in (A). Data is presented as mean ± s.e.m. (C) Western blot analysis of CaCO2 cells treated with increasing concentrations of MakA (24 h). Data are representative of two independent experiments. The numbers below indicates quantification of MakA relative to actin. (D-E) CaCO2 cells were treated with increasing concentrations of Alexa568-MakA (24 h). Flow cytometry analysis indicates concentration dependent increase in cellular uptake of Alexa568-MakA. Data points represents five biologically independent experiments; bar graphs show mean ± s.d. Significance was determined from biological replicates using a one-way analysis of variance (ANOVA) with Dunnett’s post-test against vehicle control. *p<0.05, **p<0.01. (F) Quantification of Western blot of CaCO2 cells treated with MakA (250 nM) for 24 h with or without the inhibitors; LY294002 (20 μM) or nocodazole (100 nM). The histogram represents quantification of MakA from three biologically independent experiments; bar graphs show mean ± s.d. Significance was determined from biological replicates using a one-way analysis of variance (ANOVA) with Sidak’s multiple comparisons test against MakA detected in the membrane fraction of the cell. *p<0.05, **p<0.01. (G) HCT8 cells were treated with MakA (250 nM) for 24 h. Cellular localization of MakA was detected using anti-MakA antiserum, while actin filaments were stained with FITC-labelled Phalloidin. Arrowheads indicate colocalization of MakA (red) and actin filaments (green). Nucle [file ppat.1009414.s008.tif]

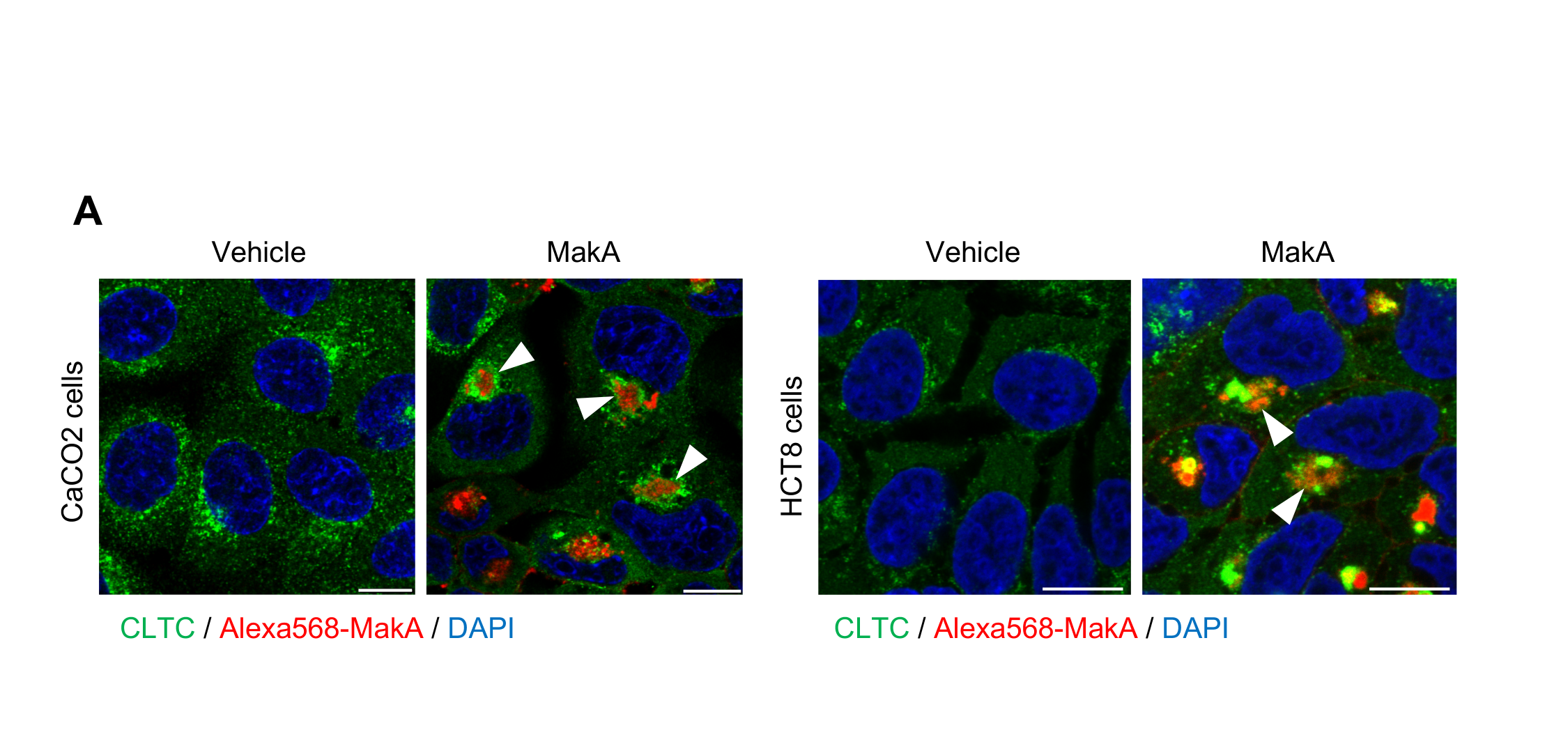

Supplement: S7 Fig — CaCO2 or HCT8 cells treated with Alexa568-MakA (250 nM) for 24 h. Clathrin was detected using anti-clathrin heavy chain antibody (CLTC). Arrowhead indicates accumulation of CLTC (green) around the perinuclear aggregate of Alexa568-MakA (red). Nuclei were counterstained with DAPI. Scale bar, 10 μm. (TIF) [file ppat.1009414.s009.tif]

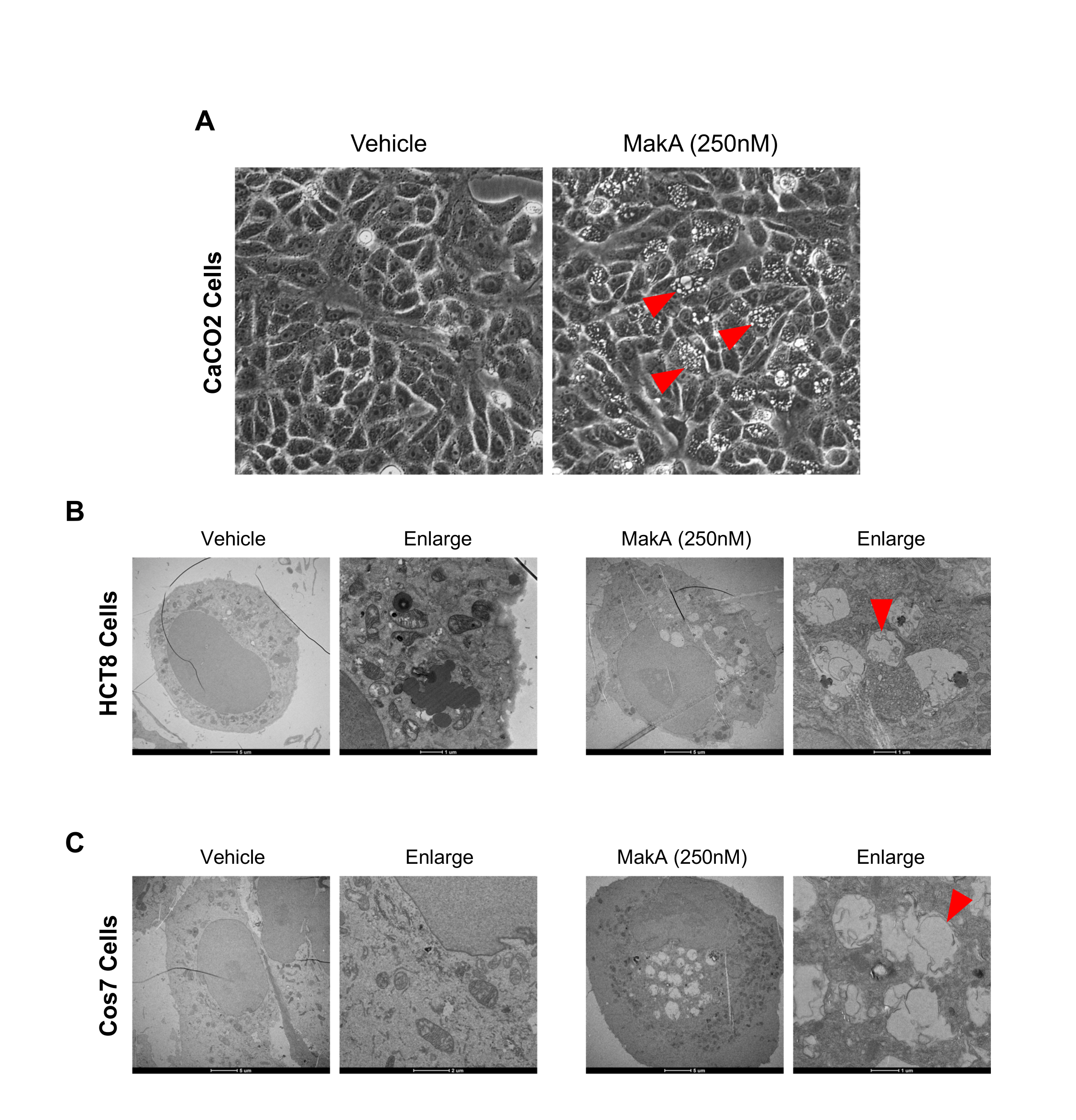

Supplement: S8 Fig — (A) CaCO2 cells treated with MakA (250 nM) for 24 h were visualized by phase contrast microscopy. Arrowhead (red) indicates vacuolation of the CaCO2 cells in response to MakA. (B-C) Representative electron micrographs of vehicle and MakA (24 h, 250 nM) treated HCT8 and Cos7 cells. Scale bar, 5 μm. Arrowhead (red) in the right panel indicates the presence of membranes inside the vacuoles of HCT8 and Cos7 cells. Scale bar, 1 μm. (TIF) [file ppat.1009414.s010.tif]

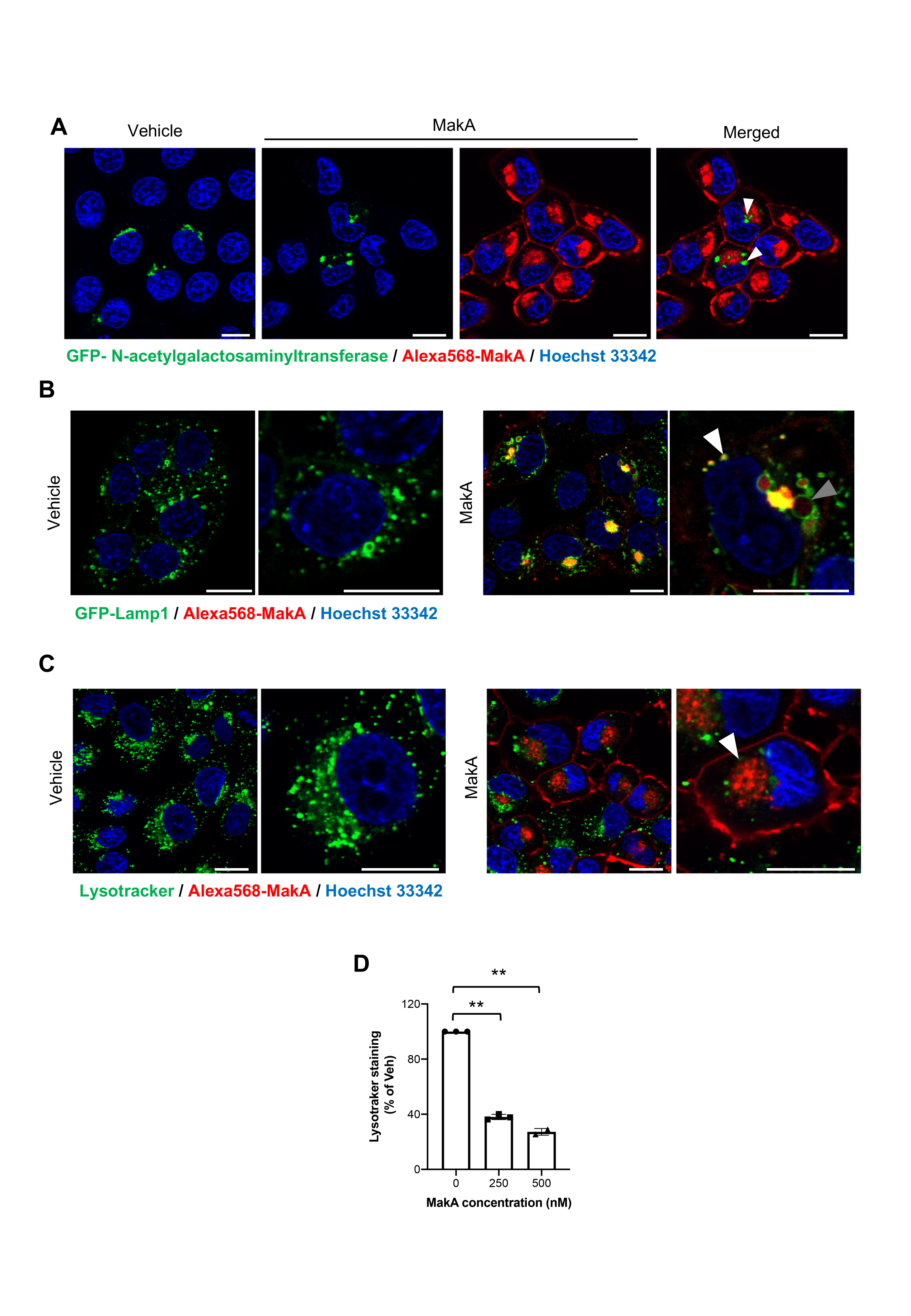

Supplement: S9 Fig — (A) CaCO2 cells transfected with Cell Light Golgi-GFP were treated with vehicle or 250 nM Alexa568-MakA for 24h. Arrowhead (white) indicates Golgi fragmentation in Alexa568-MakA treated cells. Nuclei were counterstained with Hoechst 33342. Scale bars, 10 μm. (B) CaCO2 cells transfected with Cell Light Lysosomes-GFP (24 h) and treated with Vehicle (Tris 20 mM) or Alexa568MakA (250 nM) for 24h. Arrowhead (white) indicate lysosomes stained positive for GFP-Lamp1 and Alexa568MakA, while arrowhead (gray) indicates enlargement of lysosomes. Nuclei were counterstained with Hoechst 33342. Scale bars, 10 μm. (C) CaCO2 cells treated with vehicle or Alexa568-MakA (red, 250 nM) for 24h. Cells were counterstained with Lysotracker (green, 500 nM, 30 min). Nuclei were counterstained with Hoechst 33342. Arrowhead indicates loss of lysotracker staining in MakA positive cells. Scale bars, 10 μm. (D) HCT8 cells were treated with increasing concentrations of MakA (24 h) and stained with Lysotracker (500nM, 30 min). Flow cytometry analysis indicates concentration dependent decrease in lysotracker staining. Data points represents two to three biologically independent experiments; bar graphs show mean ± s.d. Significance was determined from biological replicates using a one-way analysis of variance (ANOVA) with Sidak’s multiple comparisons test against Vehicle (Veh) treated cell. **p<0.01. (TIF) [file ppat.1009414.s011.tif]

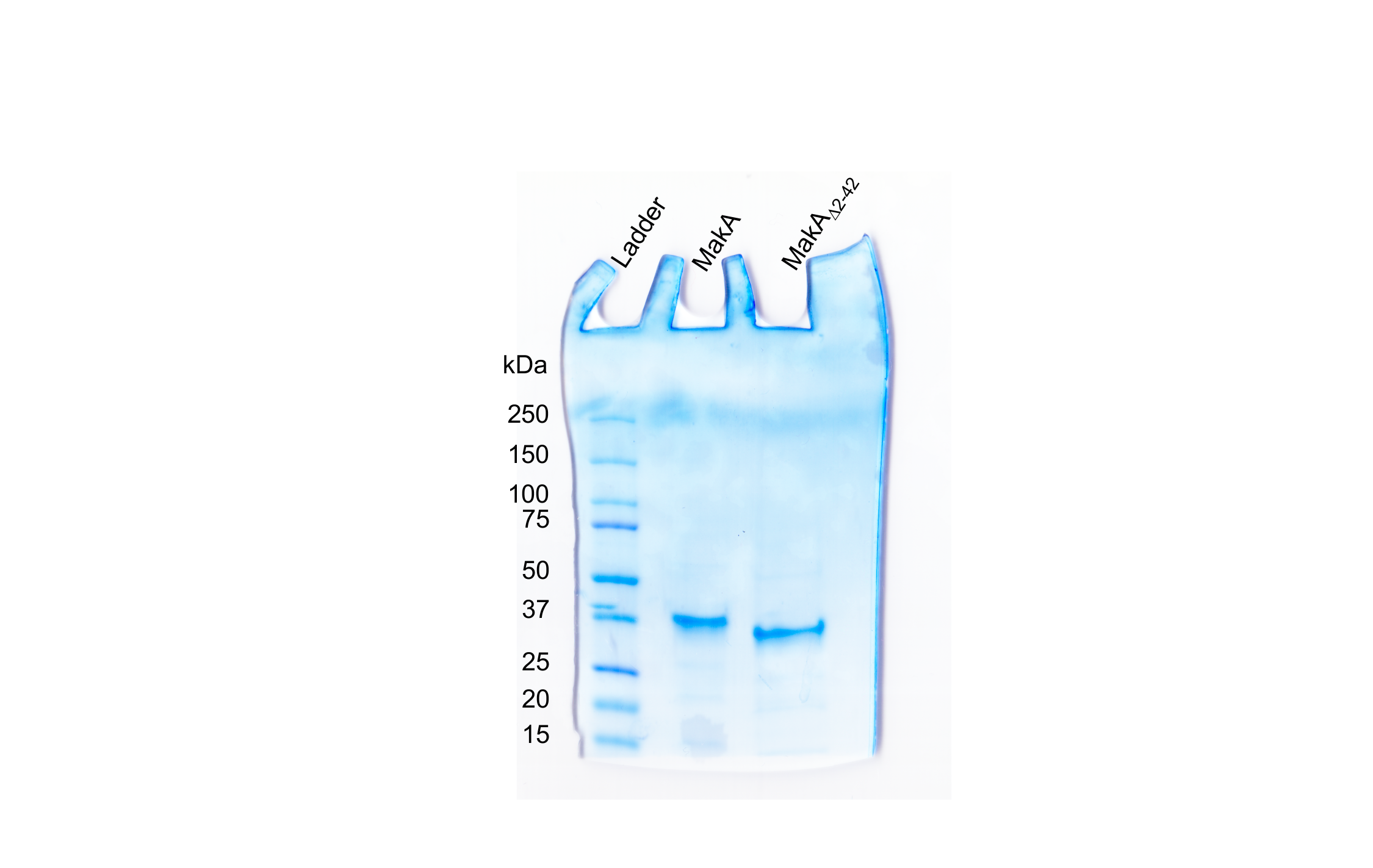

Supplement: S10 Fig — The MakA and MakAΔ2–42 proteins were purified as described in the materials and methods. The protein samples were run on a SDS-PAGE and the gel was stained with Coomassie blue stain. (TIF) [file ppat.1009414.s012.tif]
