# Supplementary material for: Phosphatidic acid-mediated binding and mammalian cell internalization of the Vibrio cholerae cytotoxin MakA
Source: PLoS Pathog. 2021 Mar 18;17(3):e1009414. doi: 10.1371/journal.ppat.1009414 (PMC8009392; doi:10.1371/journal.ppat.1009414)
Supplement: S1 Table — (DOCX) [file ppat.1009414.s013.docx]

**S1 Table. Conformational analysis of wild-type MakA and the MakA truncated variant, MakA_∆2-42_ for structural integrity by CD analysis**

|  | Helix | Antiparallel | Parallel | β-turn | Random Coil |
| --- | --- | --- | --- | --- | --- |
| MakA | 73.1 % | 0.4 % | 4.7 % | 9.4 % | 12.5 % |
| MakA_∆2-42_ | 74.6 % | 0.4 % | 4.8 % | 9.2 % | 12.8 % |
