# Supplementary material for: Phosphatidic acid-mediated binding and mammalian cell internalization of the Vibrio cholerae cytotoxin MakA
Source: PLoS Pathog. 2021 Mar 18;17(3):e1009414. doi: 10.1371/journal.ppat.1009414 (PMC8009392; doi:10.1371/journal.ppat.1009414)
Supplement: S2 Table — (DOCX) [file ppat.1009414.s014.docx]

**S2 Table: Bacterial strains and plasmids used in this study**

| Strain/  plasmid | Description/relevant characteristics | Reference / Source |
| --- | --- | --- |
| ***Vibrio cholerae*** | | |
| *V. cholerae* A1552 | El Tor, Inaba, RifR | [1] |
| MDS005 | A1552 Δ*makA* | [2] |
| SM01 | A1552 Δ*hapA* | This Study |
| SM02 | A1552 Δ*hapA*Δ*makA* | This Study |
| ***Escherichia coli*** | | |
| DH5∝ | F- Φ80lacZΔM15 Δ(*lac*ZYA-argF)  U169 *recA*1 *end*A1 *hsd*R17(rk-, mk+) *phoA* *sup*E44 *thi*-1 *gyr*A96 *rel*A1 λ- | [3] |
| BL21(DE3) | *fhuA*2 [ lon] *ompT* *gal* (λ DE3) [dcm] Δ *hsd*S  λ DE3 = λ s BamHIo Δ EcoRI-B int::  ( *lac*I::P*lac*UV5:: T7gene1) i21Δ *nin*5 | [4] |
| **Plasmids** |  |  |
| pCVD442  pCVD*hapA*  pEGFP-C1  pEGFP-N1  pEGFP-N1  CellLight  Golgi-GFP  CellLight Lysosomes-GFP | Cb^R^-positive selection suicide vector plasmid  pCVD442-based suicide plasmid for generating Δ*hapA*  To generate enhanced green fluorescent protein-PA biosensor with superior sensitivity (EGFP-PASS), a nuclear export sequence (NES) derived from protein kinase A inhibitor α (PKI-α) (26) was added between EGFP and Spo20-PABD cloned in pEGFP-C1. PASS tagged with monomeric GFP or RFP (mGFP or mRFP) was generated by replacing EGFP in the EGFP-PASS with mGFP or mRFP using AgeI and BsrGI sites.  pEGFP-N1 based expression vector used for overexpression of Cav1-GFP  pEGFP-N1 based expression vector used for overexpression of GFP-Dynamin 2 K44A  CellLight Golgi-GFP, BacMam 2.0, is a fusion construct of human golgi resident enzyme (N-acetylgalactosaminyltransferase) and emGFP.  CellLight Lysosomes-GFP, BacMam 2.0, is a fusion construct of Lamp1 (lysosomal associated membrane protein 1) and emGFP. | [5]  [6]  [7]  [8]  [9]  Invitrogen  Invitrogen |

**References**

1. Yildiz FH, Schoolnik GK. Role of rpoS in stress survival and virulence of Vibrio cholerae. J Bacteriol. 1998;180(4):773-84. Epub 1998/02/24. doi: 10.1128/JB.180.4.773-784.1998. PubMed PMID: 9473029; PubMed Central PMCID: PMCPMC106954.

2. Dongre M, Singh B, Aung KM, Larsson P, Miftakhova R, Persson K, et al. Flagella-mediated secretion of a novel Vibrio cholerae cytotoxin affecting both vertebrate and invertebrate hosts. Commun Biol. 2018;1:59. Epub 2018/10/03. doi: 10.1038/s42003-018-0065-z. PubMed PMID: 30271941; PubMed Central PMCID: PMCPMC6123715.

3. Grant SG, Jessee J, Bloom FR, Hanahan D. Differential plasmid rescue from transgenic mouse DNAs into Escherichia coli methylation-restriction mutants. Proc Natl Acad Sci U S A. 1990;87(12):4645-9. Epub 1990/06/01. doi: 10.1073/pnas.87.12.4645. PubMed PMID: 2162051; PubMed Central PMCID: PMCPMC54173.

4. Studier FW, Moffatt BA. Use of bacteriophage T7 RNA polymerase to direct selective high-level expression of cloned genes. J Mol Biol. 1986;189(1):113-30. Epub 1986/05/05. doi: 10.1016/0022-2836(86)90385-2. PubMed PMID: 3537305.

5. Donnenberg MS, Kaper JB. Construction of an eae deletion mutant of enteropathogenic Escherichia coli by using a positive-selection suicide vector. Infect Immun. 1991;59(12):4310-7. Epub 1991/12/01. doi: 10.1128/IAI.59.12.4310-4317.1991. PubMed PMID: 1937792; PubMed Central PMCID: PMCPMC259042.

6. Vaitkevicius K, Lindmark B, Ou G, Song T, Toma C, Iwanaga M, et al. A Vibrio cholerae protease needed for killing of Caenorhabditis elegans has a role in protection from natural predator grazing. Proc Natl Acad Sci U S A. 2006;103(24):9280-5. Epub 2006/06/07. doi: 10.1073/pnas.0601754103. PubMed PMID: 16754867; PubMed Central PMCID: PMCPMC1482601.

7. Zhang F, Wang Z, Lu M, Yonekubo Y, Liang X, Zhang Y, et al. Temporal production of the signaling lipid phosphatidic acid by phospholipase D2 determines the output of extracellular signal-regulated kinase signaling in cancer cells. Mol Cell Biol. 2014;34(1):84-95. Epub 2013/10/30. doi: 10.1128/MCB.00987-13. PubMed PMID: 24164897; PubMed Central PMCID: PMCPMC3911278.

8. Tagawa A, Mezzacasa A, Hayer A, Longatti A, Pelkmans L, Helenius A. Assembly and trafficking of caveolar domains in the cell: caveolae as stable, cargo-triggered, vesicular transporters. J Cell Biol. 2005;170(5):769-79. Epub 2005/09/01. doi: 10.1083/jcb.200506103. PubMed PMID: 16129785; PubMed Central PMCID: PMCPMC2171342.

9. Ochoa GC, Slepnev VI, Neff L, Ringstad N, Takei K, Daniell L, et al. A functional link between dynamin and the actin cytoskeleton at podosomes. J Cell Biol. 2000;150(2):377-89. Epub 2000/07/26. doi: 10.1083/jcb.150.2.377. PubMed PMID: 10908579; PubMed Central PMCID: PMCPMC2180219.
